# Investigation of Biotransformation Pathways in a Chimeric Mouse with a Humanized Liver

**DOI:** 10.3390/ijms26031141

**Published:** 2025-01-28

**Authors:** Isabella B. Karlsson, Anja Ekdahl, Hugh Etchingham-Coll, Xue-Qing Li, Cecilia Ericsson, Marie Ahlqvist, Kristin Samuelsson

**Affiliations:** Drug Metabolism and Pharmacokinetics, Research and Early Development, Cardiovascular, Renal and Metabolism, BioPharmaceuticals R&D, AstraZeneca, 431 83 Gothenburg, Sweden; anja.ekdahl1@astrazeneca.com (A.E.); hughcoll07@gmail.com (H.E.-C.); xueqing.li@astrazeneca.com (X.-Q.L.); cecilia.ericsson@astrazeneca.com (C.E.); marie.ahlqvist@astrazeneca.com (M.A.)

**Keywords:** biotransformation, drug metabolism, in vitro, in vivo, hepatocytes, PXB-mouse^®^

## Abstract

Xenobiotics, including drugs, undergo metabolism to facilitate detoxification and excretion. Predicting a compound’s metabolic fate before clinical trials is crucial for efficacy and safety. The existing methods rely on in vitro systems and in vivo animal testing. In vitro systems do not replicate the complexity of in vivo systems, and differences in biotransformation pathways between humans and nonclinical species may occur; thus, accurate predictions of human-specific drug metabolism are not always achieved. The aim of this study was to evaluate whether a chimeric mouse with a humanized liver, specifically the PXB-mouse, can mimic human metabolic profiles. PXB-mice have livers engrafted with up to 95% human hepatocytes. The biotransformation of 12 different small-molecule drugs were evaluated in PXB-mice (through analysis of blood and urine) and compared with the metabolism by hepatocytes from humans and mice and, when available, literature reports on human in vivo metabolism. The detected metabolites included major Phase I and II transitions, such as hydroxylation, and N- and O-dealkylation and glucuronidation. The metabolic patterns of the PXB-mice closely matched human in vivo data. It is also worth noting that the human hepatocytes formed most of the circulating metabolites, indicating that hepatocytes provide reliable predictions of human metabolic pathways. Thus, for drugs with human biotransformation pathways that are not observed in nonclinical species, the PXB-mouse model can be valuable in predicting human-specific metabolism.

## 1. Introduction

When entering the body, xenobiotics, including drugs, are normally subjected to metabolism to facilitate their removal via detoxification and excretion. It is desirable, for both efficacy and safety, to be able to prospectively determine a compound’s metabolic fate prior to administration during clinical trials. The existing methods for preclinical testing of drug metabolism rely on in vitro systems and in vivo animal testing. Due to deficiencies in replicating complex in vivo metabolism using in vitro methods, and differences in biotransformation in nonclinical species versus humans [1], which themselves are only partially understood, accurate prediction of human-specific drug pathways is not always attainable [2,3,4]. Interspecies differences in drug metabolism produce both quantitative and qualitative differences in the metabolites produced by human and animal species. This is a problematic issue in the preclinical evaluation of drugs due to the inability to identify human-specific and human-predominant metabolites, if present, as they may be the source of potentially adverse events or drug-induced toxicity [5,6]. If a candidate drug has either human-specific or human-predominant metabolites, the use of nonclinical animal testing for toxicity aspects is quite limited. This has led to the development of a chimeric mouse with a humanized liver, which expresses Phase I and II human-specific enzymes, along with transporter proteins that display a similar profile as those found in the hepatocytes of the human donor [7,8,9,10,11,12,13,14,15]. Humanized liver chimeric mice are produced by transplanting healthy human hepatocytes into genetically modified immunodeficient mice with liver injuries [16]. Previous research demonstrated that humanized liver chimeric mice derived from albumin enhancer/promoter-driven cDNA of urokinase-type plasminogen activator/severe combined immunodeficiency (cDNA-uPA/SCID) mice maintained stable human hepatocyte populations for several months [17]. The hepatocytes constitute a mature organ, with human specific metabolism and excretion pathways, along with an array of enzymes matching those of the donor [18,19]. This is a potentially valuable in vivo model for the study of human metabolism.

**Figure 1:**
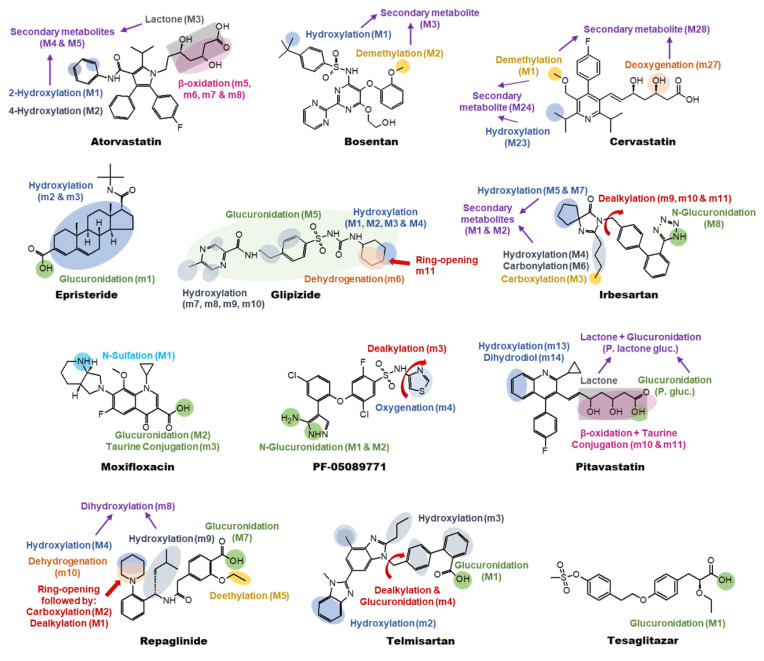
Structure and metabolic transformations of the investigated compounds.

The aim of this study was to evaluate the utility of chimeric mice with humanized livers in predicting human drug metabolism. Specifically, we used the commercially available PXB-mouse model [20], which contains up to 95% human hepatocytes, to explore its potential for predicting circulating and renally excreted metabolites of administered drugs. Additionally, the metabolites formed from the same set of drugs were assessed in vitro in human and mouse hepatocytes. The circulating and renally excreted metabolites in the PXB-mice were compared with the metabolites detected in the hepatocytes and to human in vivo literature data (see Figure S1 for a schematic presentation of the workflow). The compounds investigated were atorvastatin, bosentan, cerivastatin, epristeride, glipizide, irbesartan, moxifloaxcin, PF-05089771, pitavastatin, repaglinide, telmisartan, and tesaglitazar (see Figure 1).

## 2. Results and Discussion

The investigated compounds were administered via an intravenous bolus tail vein injection and cassette dosing was used in order to minimize the number of animals needed for the study. Each cassette contained three different compounds (0.5 mg/kg/test compound) and each group contained three PXB-mice. Blood was collected after 5 min, 10 min, 30 min, 1 h, 3 h, 8 h, and 24 h post-dose. Urine was collected at 0–4 h, 4–8 h, and 8–24 h. The replacement index (RI), the percentage of human hepatocytes out of all the hepatocytes in the liver, was determined by measuring the human albumin (hAlb) levels in the blood [16] and was determined to be >85% for the animals used.

Whole blood extracts and urine from the PXB-mice were profiled for the presence of the parent drugs and their respective metabolites in circulation using qualitative liquid chromatography coupled to high-resolution mass spectrometry (LC-HRMS). The selected drugs were also evaluated in vitro in human and mouse hepatocytes. The human hepatocytes used came from a mixed gender pool of 10 donors and were not the same as the single donor used for the PXB-mice. The hepatocytes were incubated with the selected compounds for 120 min. In addition to the final incubation time point, samples were also taken at 0 min and after 40 min. Literature reports of human in vivo metabolism were compared to the metabolic profiles seen in the PXB-mouse blood and urine samples and in the human and mouse hepatocytes. Throughout the article, the reported human in vivo metabolites are denoted with a capital M (e.g., M1) and metabolites only detected in vitro or in vivo in animals are denoted with a small m (e.g., m6).

### 2.1. Atorvastatin

In humans, atorvastatin is primarily metabolized by cytochrome P450 enzymes (CYP), particularly CYP3A4, through aromatic 2- and 4-hydroxylation and lactonization (M1 to M5). The major elimination route of the administered drug is via biliary excretion, rather than through renal excretion (<1% of the administered dose) [21]. In mice, however, different metabolic pathways were observed, with β-oxidation being the primary route [22]. This result aligns with the in vitro metabolites that were detected in the mouse hepatocytes in this study (Figure 1 and Table 1). Conversely, the metabolite profiles obtained from the samples from the PXB-mice showed similar metabolic pathways to humans, with 2-hydroxylation and lactonization (M2 and M5) being dominant (Figure S2). All human circulating metabolites were also detected in the human hepatocyte incubations. Additionally, the major β-oxidized mouse metabolite m6 was also detected in the human hepatocytes; however, it was not reported in humans in vivo. In summary, all human metabolites observed in vivo were accounted for in both the human hepatocyte incubations and PXB-mouse studies.

### 2.2. Bosentan

In a clinical study, more than 97% of the administrated radioactive dose was recovered following both oral and intravenous administrations of bosentan, with over 90% recovered in the feces and less than 6% in the urine [23]. The primary metabolite detected in the plasma, urine, and feces was Ro 48-5033 (M1), which resulted from the hydroxylation of the tert-butyl group (Table 1 and Figure S3). Metabolite Ro 64-1056 (M3), a secondary metabolite which corresponds to hydroxylation of the tert-butyl group and demethylation of the phenolic methyl-ester, was the second major metabolite detected in the urine and feces. Interestingly, this metabolite was not observed in the plasma. Metabolite Ro 47-8634 (M2), formed after the demethylation of the phenolic methyl-ester, represented only a minor metabolite fraction in all the matrices investigated. Bosentan is mainly metabolized in the liver by the cytochrome P450 enzymes CYP2C9 and CYP3A4 [32], and the formation of the phenol metabolite Ro 47-8634 (M2) is only metabolized by CYP 3A4, while both the hydroxylated metabolite Ro 48-5033 (M1) and the secondary metabolite Ro 64-1056 (M3) are metabolized via CYP3A4 and CYP2C9. The metabolic conversion of bosentan was significantly higher in the mouse hepatocytes compared to the human hepatocytes and 63% (relative amount) and 94% (relative amount) remained of the parent compound at the 120 min time point, respectively. In addition to metabolites M1, M2, and M3, three additional metabolites (m4, m5, and m6) were formed in the mouse hepatocytes (Figure 1 and Figure S3 and Table 1). The secondary metabolite m4 was proposed to be formed after double oxidation and dehydrogenation, while metabolite m5 was proposed to be formed after hydroxylation and conjugation with glucuronic acid. Metabolite m6 was detected as a second hydroxylated metabolite. In the mouse hepatocytes, the major metabolites detected were metabolites M1 and m4. In the human hepatocytes, the major metabolite detected was metabolite M1 in addition to the two minor metabolites M2 and M3. In the urine from the PXB-mice, metabolites M1, M2, and M3 as well as metabolite m4 were detected while metabolite M1 was the only metabolite detected in the PXB-mouse blood. Metabolite M1 was by far the most abundant metabolite detected in the urine from the PXB-mice. Notably, the comparison of metabolites detected in the human hepatocyte incubations closely reflected those reported in human in vivo studies, as did the metabolites found in the urine from the PXB-mice.

### 2.3. Cerivastatin

The metabolic profile of cerivastatin has been extensively investigated through both in vitro and in vivo studies in human and animal models [24,33,34,35]. In human in vivo studies, two primary oxidative biotransformation pathways were identified: demethylation (M1) and hydroxylation of the isopropyl substituent (M23) [24]. A combination of these reactions (M24) was observed in the excreta but was absent in human plasma (see Figure 1 and Figure S4 and Table 1). In vivo studies in mice highlighted two major plasma metabolites: deoxygenation (m27) and a combination of deoxygenation with demethylation (m28) metabolites [33]. Notably, the main metabolites in human plasma, M1 and M23, were not detected in the mouse in vivo samples. In both humans and mice, only minor quantities of metabolites were excreted via urine.

In the human hepatocytes, the parent compound M1, M23, and minor quantities of M24 were observed (Table 1). In contrast, the mouse hepatocytes predominantly produced m27 and m28, with smaller amounts of M1, M23, and M24 also present. In the blood samples from the PXB-mice, the parent compound, along with human metabolites M1 and M23 and mouse-specific metabolites m27 and m28, were identified. The urine from the PXB-mice primarily contained human metabolites M1 and M23, along with minor amounts of the parent compound and m28. This suggests that PXB-mice exhibit a metabolic profile more closely aligned with humans compared to mice, while still retaining mouse-specific metabolites.

### 2.4. Epristeride

Epristeride was found to be metabolically stable in the PXB-mice as well as in the human and mouse hepatocytes. The only detected drug-related material in the PXB-mouse blood was epristeride itself and in the PXB-mouse urine, a 4:1 mixture (relative MS area) of epristeride and the acylglucuronide was detected (Figure 1 and Figure S5 and Table 1). Over 90% (relative amount) of the epristeride was present in the human hepatocytes after 120 min of incubation and the only metabolite present at >1% was the acylglucuronide. In addition, low amounts of two different aliphatic hydroxylation metabolites (m2 and m3) could be seen (Figure 1 and Figure S5 and Table 1). The only detected metabolite in the mouse hepatocytes after 120 min of incubation was the acylglucuronide. To the best of our knowledge, no human in vivo data have been reported for epristeride.

### 2.5. Glipizide

Glipizide, belonging to the sulfonylurea derivative class of compounds, has been the subject of several clinical studies [36,37,38]. The major route of elimination for glipizide was renally, with >65% of the radioactive dose recovered in urine. Glipizide is mainly metabolized by hepatic CYP2C9 and only a minor part of the radioactivity in urine was unchanged glipizine. The two major metabolites detected in urine were the 3-cis-hydroxycyclohexyl (M3) and the 4-trans-hydroxycyclohexyl (M1) derivatives (Figure 1 and Figure S6 and Table 1). In the urine samples, one metabolite was characterized as N-(2-acetylamino-ethyl-phenyl-sulfonyl)-N’-cyclohexyl urea (DCDA), and the rest were unidentified metabolites [36,38]. In the plasma samples, glipizide was the major component detected, together with hydroxylated metabolites, which were suggested to be one or both the 3-cis-hydroxycyclohexyl and 4-trans-hydroxycyclohexyl derivatives. In addition, a glucuronidated metabolite along with non-extractable metabolites were reported. A more recent study in healthy volunteers published in 2017 described the identification and quantification of four hydroxylated metabolites of glipizide in urine [25]. In addition to the previously reported hydroxylated metabolites, they identified and quantified the 4-cis-hydroxycyclohexyl (M2) and the 3-trans-hydroxycyclohexyl (M4) derivatives. 

In the human and mouse hepatocyte incubations as well as in the blood and urine from the PXB-mice, multiple hydroxylated metabolites were found (Figure 1 and Figure S6 and Table 1). The turnover of glipizide in the hepatocyte incubations was lower in the human than in the mouse hepatocytes and 92% (relative amount) and 85% (relative amount) of the parent compound remained at the 120 min time point, respectively, which could explain the smaller number of metabolites detected in the human hepatocyte incubations. In the hepatocytes as well as in the blood and urine from the PXB-mice, metabolites M1, M2, M3, and M4 were detected. In the urine from the PXB-mice and in the mouse hepatocytes, four additional hydroxylated metabolites were detected (m7, m8, m9, and m10). A secondary metabolite corresponding to a double oxidation and dehydrated metabolite (m11) was detected in the mouse hepatocytes. In the PXB-mouse blood, one metabolite corresponding to a dehydrogenated compound (m6) and one metabolite corresponding to a glucuronic acid conjugate (M5) were also detected (Figure 1 and Figure S6). The dehydrogenated metabolite m6, suggested to be from a dehydrogenation of the cyclohexyl ring, was the major metabolite formed in the PXB-mouse blood, followed by the hydroxylated metabolites M1, M2, M3, and M4. The most abundant metabolites in the PXB-mouse urine were the hydroxylated metabolites M1, M2, M3, and M4. The DCDA metabolite reported to be found in trace levels in human urine [36,38] was not detected in the urine or blood from the PXB-mice nor in the mouse or human hepatocyte incubations. In summary, the human hepatocyte and PXB-mouse results reflected the metabolites formed and reported in human in vivo studies. 

### 2.6. Irbesartan

Chando et al. investigated the biotransformation of irbesartan in healthy human subjects using radiolabeled materials [26]. They quantified and characterized eight metabolites of irbesartan in human plasma and urine, including a tetrazole N^2^-β-glucuronide conjugate (M8), monohydroxylated metabolites from the butyl side chain (M4) and spirocyclopentane ring (M5 and M7), a diol (M1), a keto metabolite (M6), a keto-alcohol (M2), and a carboxylic acid metabolite (M3) (Figure 1 and Figure S7 and Table 1).

In plasma, irbesartan was the predominant component, with none of the metabolites exceeding 9% of the plasma radioactivity. Approximately 20% of the radiolabeled dose was recovered in urine, with the ω-1 hydroxylated metabolite (M4) being the predominant metabolite excreted [26].

All human urine metabolites were detected in the PXB-mouse urine and all human plasma metabolites above 1% (M4-M8) were detected in the PXB-mouse blood. In addition to the human in vivo metabolites, major N-dealkylation metabolites (m9-m11) were seen in the PXB-mouse blood (m9–m11) and urine (m9 and m11). In the human in vivo study by Chando et al. [26], they noted that there were unidentified metabolites in the urine that eluted earlier than the identified metabolites. The authors stated that these metabolites are likely to be N-dealkylation metabolites; however, as the total radioactivity of the unidentified metabolites was approximately 10% of the radioactivity in urine, the authors concluded that they were minor metabolites and therefore structural elucidation was not deemed necessary. Metabolites M1-M3 and M6 were not detected in the human hepatocytes (Figure S7 and Table 1). The only detected metabolites were M4, M5/M7, and M8.

The metabolic conversion of irbesartan was significantly higher in the mouse hepatocytes compared to the human hepatocytes, and only 2.4% (relative amount) remained of the parent compound in the mouse hepatocytes at the 40 min time point. The human in vivo metabolites M1, M3, and M4-M7 were detected in the mouse hepatocytes. The secondary metabolite M2 was not detected, and neither was the direct glucuronidation metabolite M8; however, glucuronide conjugates of M4 and M5/M7 were seen in the mouse hepatocytes. In addition to the human in vivo metabolites, large amounts of N-dealkylation metabolites (m9-m11) were detected in the mouse hepatocytes (Figure S7 and Table 1).

In summary, the PXB-mouse generated a human-like metabolic pattern for irbesartan, with coverage of all human urine metabolites and all circulating metabolites above 1%. One could argue that the presence of major N-dealkylation metabolites in both the blood and urine of the PXB-mice is a result of mouse biotransformation pathways. However, as the current study has only recorded relative amounts (assuming similar ionization efficiencies between the parent drug and its metabolites) and that Chando et al. [26] report unidentified urine metabolites eluting early in the chromatograms and hypothesized that they are N-dealkylation metabolites, we cannot say for certain that there is a difference in the amounts of N-dealkylation metabolites formed in vivo between humans and PXB-mice. It is also worth noting that although the human hepatocytes failed to form four out of the eight reported human in vivo metabolites, the combination of human and mouse hepatocytes would have detected all human in vivo metabolites except M2. Considering that M2 is a downstream metabolite of M4, which was detected in both the human and mouse hepatocytes, all human metabolic pathways were covered by the hepatocytes (Figure S7 and Table 1), providing satisfactory coverage of the human in vivo metabolites.

### 2.7. Moxifloxacin

The metabolism of moxifloxacin in humans was reported to be mainly via Phase 2 metabolic conjugations with no P450 involved [27]. Two metabolites were identified as N-sulphate (M1) and acylglucuronide (M2), both of which were found in human plasma and urine (Figure 1 and Figure S8 and Table 1). In this study, M1 and M2 were identified as the main metabolites in the human and mouse hepatocyte incubations, as well as in the blood and urine samples from the PXB-mice, which aligns with the human data (Table 1). Additionally, a taurine conjugate (m3) was also detected in the mouse hepatocyte incubations as well as in the urine from the PXB-mice, which suggested the presence of acyl-CoA-mediated conjugation. In summary, the PXB-mice exhibited Phase 2 drug-metabolizing enzyme activities that closely mirror the in vivo human metabolic pathways for moxifloxacin.

### 2.8. PF-05089771

PF-05089771 was developed as a voltage-gated sodium channel blocker for use as a pain therapeutic. To the best of our knowledge, the metabolic fate of this compound has not been published. The metabolites found in the blood and urine from the PXB-mice were consistent with those observed in the human hepatocyte incubations, including two direct N-glucuronides (m1 and m2), an oxygenated metabolite (m3), and an N-dealkylated metabolite (m4) (Figure 1 and Figure S9 and Table 1). In the mouse hepatocytes, only the oxidized products m3 and m4 were detected. It is well documented that significant species differences were observed in the capacity and specificity of the N-glucuronidation metabolic pathway, and in general, N-glucuronidation reactions are much more prevalent in humans than in laboratory animals [39,40]. This study demonstrated the absence of N-glucuronide formation in the mouse hepatocyte incubations and the reintroduction of this metabolic activity in the PXB-mice. This suggests that PXB-mice can mimic human metabolic pathways to some extent, particularly in the formation of N-glucuronides. The study underscores the importance of species differences and highlights the potential of PXB-mice as a valuable model for more accurately predicting human biotransformation pathways, particularly when the prevalent metabolic reactions in humans are expected to be involved.

### 2.9. Pitavastatin

In a study by Fujino et al. [28], the metabolism of pitavastatin was studied in healthy male volunteers. The only major human metabolite detected in plasma was pitavastatin lactone (Figure 1 and Figure S10 and Table 1), which was present in high amounts. Trace amounts of a β-oxidation metabolite (M9) was detected at the later time points (6 h and 24 h). No other circulating human metabolites were reported. In urine, the excretion of unchanged pitavastatin was 0.7% and the excretion of pitavastatin lactone was 1.7% of the dose [28]. Enzymatic hydrolysis with β-glucuronidase revealed that about 4% of the dose was excreted as glucuronide conjugates of pitavastatin and pitavastatin lactone, making the glucuronide conjugates the major urine metabolites. Low levels of a dehydrolactone (M2) could also be seen [28].

The only major circulating human metabolite, pitavastatin lactone, was also the major metabolite in the PXB-mouse blood. It would appear as if the amount of unchanged pitavastatin in circulation is higher in the PXB-mice compared to humans. However, as the current study only used relative amounts and assumed similar ionization efficiencies, it cannot be excluded that the apparent difference is due to differences in ionization efficiencies between pitavastatin and the lactone. Pitavastatin lactone glucuronide could also be detected in the PXB-mouse blood. In the PXB-mouse urine, relative amounts >10% of unchanged pitavastatin were detected together with lower levels of pitavastatin lactone and pitavastatin lactone glucuronide. The largest urine metabolite (>50% relative amount), however, was a quinoline dihydrodiol (m14) (Figure 1 and Figure S10 and Table 1), which was not seen in vivo in humans. The human hepatocytes generated a metabolic profile that was very similar to that reported in the human in vivo study (Table 1). The lactone constituted >50% (relative amount) after 120 min of incubation and low levels of the in vivo urine metabolites pitavastatin glucuronide and pitavastatin lactone glucuronide were also detected. In addition to the human in vivo metabolites, low levels of the major PXB-mouse urine metabolite, the dihydrodiol of the quinoline ring (m14), was seen together with an aromatic quinoline hydroxylation metabolite (m13) (Figure 1 and Figure S10 and Table 1). The mouse hepatocytes produced a completely different metabolic profile, with β-oxidation followed by taurine conjugation forming the major metabolites (m10 and m11). Both of these metabolites have been reported as biliary metabolites in rats in vivo [28].

An in vitro study by Fujino et al. [41] showed that active UGT is needed for the lactone to form, i.e., the glucuronidation of pitavastatin occurs first, followed by an elimination reaction that results in the lactone. Yamada et al. [42] found that in monkey microsomes, the quinoline dihydroxylation metabolite is a major metabolite. They also showed that this metabolite is formed from the lactone. They concluded that quinoline dihydroxylation (m14) and the aromatic quinoline hydroxylation (m13) are more prevalent in monkeys compared to humans. It would appear as if a similar metabolic transformation also occurs in PXB-mice. Given that the quinoline dihydroxylation metabolite is derived from the lactone, and that the lactone is formed from pitavastatin glucuronide, PXB-mice can be considered able to perform human-like biotransformations of pitavastatin. In particular, the metabolic profile of pitavastatin in the mouse hepatocytes was completely different (Table 1 and Figure S10). However, the human hepatocytes provided a metabolic profile of pitavastatin that closely resembled the reported human in vivo pattern.

### 2.10. Repaglinide

In a phase I trial published by van Heiningen et al. [29], the disposition of radiolabeled repaglinide was investigated in healthy male volunteers. The majority of the administered dose (90%) appeared in the feces, while 8% was excreted in urine. The main drug-related material in plasma was repaglinide (61%), followed by the dicarboxylic acid M2 (11%), the acyl glucuronide M7 (5.7%), and the aromatic amine M1 (3.1%) (Figure 1 and Figure S11 and Table 1). All other circulating metabolites were present at <2%. In urine, the major metabolites were unidentified polar compounds, M1 (24%) and M2 (22%) (Table 1). The amount of unchanged repaglinide in urine was 1.0%.

The circulating repaglinide-related material seen in the blood from the PXB-mice comprised mainly unchanged drug (>90%, relative amount), along with low levels of metabolites M4, M7, and m8-m10 (Figure 1 and Figure S11 and Table 1). The reported human in vivo metabolites M1, M2, and M5 were not detected in the PXB-mouse blood. The largest metabolite in the PXB-mouse blood was an oxidative dehydrogenation of the piperidine ring (m10). In the PXB-mouse urine, the major metabolites (>10%, relative amount) were M4, M7, and m8. M2 was also detected, while M1, M5, and M6 were not (Table 1). Secondary metabolites such as glucuronidation metabolites of M2, M4, and m9 were also detected in the PXB-mouse urine.

All reported human metabolites were detected in the human hepatocytes (Figure 1 and Figure S11 and Table 1). M2, M4, and M7 were the three major metabolites, which were detected in relative amounts above 10%. Metabolites M1, M5, m8-m10 were also detected (Table 1). Secondary glucuronidation metabolites similar to those detected in the PXB-mouse urine were also seen. As for irbesartan, its metabolic conversion in the mouse hepatocytes was significantly faster than in the human hepatocytes and <10% (relative amount) of the parent compound remained in the mouse hepatocytes after 40 min of incubation. All reported human in vivo metabolites were also detected in the mouse hepatocytes, with relative amounts >50% for M2 (Table 1). In addition, large amounts of secondary glucuronidation metabolites were detected in the PXB-mouse urine and mouse hepatocytes.

In summary, although the PXB-mice displayed a similar metabolic profile to that reported for humans in vivo, the best prediction of human in vivo repaglinide metabolites was obtained with the human hepatocytes. In the study by van Heiningen et al. [29], it is mentioned that major unidentified polar urine metabolite(s) were detected. Later studies [43,44] have suggested that this may be the dihydroxylated metabolite m8. Metabolite m8 was one of the three major urine metabolites in the PXB-mice and it was also detected in the human hepatocytes. The structural information for the metabolites detected by van Heiningen et al. [29] in the clinical trial is somewhat lacking and the only metabolites that are clearly described are the aromatic amine M1 and the dicarboxylic acid M2. The authors referenced an abstract by Bauer et al. [45] that describes an earlier clinical study in which the racemate of repaglinide was used. That abstract describes M7 as the acylglucuronide and M4 as the hydroxylation of the piperidine ring. A study by Gan et al. [44], in which the biotransformation of repaglinide was investigated in various in vitro systems, including human S9 fractions, fresh hepatocytes, microsomes, and recombinant CYPs, found that, in absence of cytosol, CYP3A4 gives metabolite m10, a product of the oxidative dehydrogenation (−2H) of the piperidine ring instead of the ring-opened carboxylic acid M2 (see Figure S11). However, when cytosol was added to the incubation, M2 was formed instead of the oxidative dehydrogenation metabolite. No formation of M2 was seen with cytosol alone. The probable explanation for this observation is that a cytosolic enzyme, such as aldehyde oxidase (AO), is catalyzing the oxidation of the aldehyde to the carboxylic acid (M2) and thereby pushing the equilibrium toward M2 rather than m10. In absence of these cytosolic enzymes, the formation of m10 dominates. In the PXB-mouse blood, no M2 was detected, whereas the oxidative dehydrogenation metabolite m10 was the major metabolite. M2 was, however, detected in the PXB-mouse urine (Table 1).

### 2.11. Telmisartan

Telmisartan, a bi-benzamidazole molecule, has been studied in healthy volunteers and only one metabolite, the acylglucuronide conjugate (M1), has been reported [30]. The majority of the administered radioactive dose (>97%) was excreted unchanged in feces via biliary excretion and only minor amounts were found in the urine (0.91% and 0.49% of total radioactivity after oral and IV administrations, respectively). The study concluded that the cytochrome P450 isoenzymes are not involved in the metabolism of telmisartan and only unchanged telmisartan was found in feces while the acylglucuronide conjugate was detected at low levels in plasma and urine.

The metabolic turnover of telmisartan in the human hepatocytes was significantly higher than in the mouse hepatocytes, with 41% (relative amount) and 98% (relative amount) of the parent compound remaining at the 120 min time point, respectively. Two metabolites were detected in the mouse hepatocyte incubations, which were suggested to be the acylglucuronide conjugate (M1) and a hydroxylated metabolite (m2), whereas only the acylglucuronide conjugate (M1) was detected in the human hepatocytes. In the blood from the PXB-mice, only the acylglucuronide conjugate (M1) was found. Interestingly, three metabolites were detected in the urine from the PXB-mouse: the acylglucuronide conjugate (M1), another hydroxylated metabolite of telmisartan (m3), and a conjugate with glucuronic acid in the benzimidazole part of N-dealkylated telmisartan (m4) (Figure 1 and Figure S12 and Table 1). The formation of hydroxylated metabolites in the mouse hepatocytes and in the urine from the PXB-mice indicates that cytochrome P450 isoenzymes are involved in telmisartan metabolism. In summary, the results of the incubations of human and mouse hepatocytes, along with those of the PXB-mouse study, reflected the metabolite profile observed in the human in vivo study.

### 2.12. Tesaglitazar

The metabolism of tesaglitazar has been characterized in humans in vivo, revealing a singular metabolic pathway involving the formation of acylglucuronide and its isomers, which are formed through acyl migration [31]. The investigations utilizing human and mouse hepatocytes, as well as PXB-mouse blood and urine samples, identified the parent compound as the predominant compound, with only trace levels of the acylglucuronide metabolite detected (Figure 1 and Figure S13 and Table 1).

### 2.13. General Discussion

Multiple examples exist wherein the humanized liver chimeric mouse model has demonstrably produced both primary and secondary metabolites, including human-specific and human-predominant metabolites for a variety of substrates [46,47,48]. Whilst the capability of humanized liver chimeric mice to produce human metabolites exists, it has yet to be verified whether they can be prospectively used to predict human metabolite patterns and biotransformation pathways, and thus the model remains unvalidated. This aspect limits the usefulness of such a method in determining the metabolic profiles of candidate drugs in nonclinical testing. Studies have shown mixed results when using this model for predicting metabolic profiles [49]. Thus, its ability to prospectively evaluate drug candidates’ pharmacokinetics, drug–drug interactions, and metabolic profiles are currently uncertain.

This study is a continuation of our previous work on evaluating the usefulness of PXB-mice for predicting human drug metabolism. In a study by Samuelsson et al., the pharmacokinetics and biotransformation of midazolam was investigated in the PXB-mouse model and compared to that of the severe combined immunodeficient (SCID) mouse model [48]. The findings from that study were that all major circulating human metabolites were detected in the blood from the PXB-mice, whereas only 50% of the major metabolites could be detected in the SCID mice. This work was followed by an investigation of fenclozic acid, a non-steroid anti-inflammatory drug (NSAID), that was withdrawn from late-stage clinical development in patients in 1970 due to observed toxicity in human subjects, such as severe adverse drug reactions [50] and jaundice [51]. As no toxicity had been observed in animals, Ekdahl et al. investigated the metabolism of fenclozic acid in PXB-mice in order to identify human-specific metabolites that could explain the toxicity observed in humans [52]. Indeed, a number of “human”-specific metabolites were detected that might be the cause of the drug-induced liver injury observed in humans but not animals. These two studies have shown that the PXB-mouse model can provide useful insights into human drug metabolism.

In order to obtain a more complete picture of the ability of PXB-mice to predict human in vivo metabolism, in the current study, we analyzed a larger set of small-molecule drugs, which are known to be metabolized via different Phase I and II pathways. For comparison, the biotransformation of these compounds was also assessed in human and mouse hepatocytes. A summary of the different model systems’ ability to predict the human circulating and renally excreted metabolites are shown in Table 2 and Table 3, respectively. The result is highlighted in green if the model system identified more than 75% of the reported human in vivo metabolites (including all major circulating or renally excreted metabolites). If the portion of identified human in vivo metabolites was between 50 and 75%, it is highlighted in amber and when less than 50% of the reported human in vivo metabolites were identified, it was indicated by red. In general, the metabolites identified in the blood and urine from the PXB-mice corresponded with the reported human in vivo metabolites. For those compounds where a clear difference was observed in the metabolic pathways between human and mice, the PXB-mice displayed a more human-like profile, such as for atorvastatin, pitavastatin, and PF-05089771. For some compounds, a mixture of human-specific and mouse-specific metabolites were identified, such as for cerivastatin and irbesartan. The metabolite patterns in the urine from the PXB-mice aligned better with the literature than the profiles for circulating metabolites (compare Table 2 and Table 3). One explanation for this outcome could be that for many of the investigated compounds, the levels of drug-related materials in the blood from the PXB-mice were low and some metabolites may have been below the limit of detection. Another conclusion from this study is that the human hepatocytes were able to form >75% (including all major metabolites) of both the circulating and the renally excreted human metabolites for all compounds (where human in vivo metabolism was known) except for irbesartan, as shown in Table 2 and Table 3.

The main advantage of the PXB-mouse model is that it combines a human in vitro system (human hepatocytes) with an in vivo system (mouse). However, for most applications, the potential variability in CYP and transporter expression that arises from the fact that a single donor is used for the transplanted hepatocytes is a disadvantage. The natural polymorphisms of drug-metabolizing enzymes and transporter proteins in the liver of the PXB-mouse only allow for a narrow representation of the variability encountered in humans in vivo. In the current study, different numbers of hepatocyte donors were used for the generation of the PXB-mice (single donor) and the hepatocyte incubations (gender-mixed pool of donors); however, the same donor hepatocytes could be used for both engraftments in vivo and for in vitro assays in order to maximize the consistency between the two studies. The lack of repeatability, along with the potential for mouse-type metabolites to be formed, can give an unclear picture with regard to the metabolic profile of a drug. For exploratory investigations where human data are not available, such as in the case of fenclozic acid [52], or when large species differences are expected, the PXB-mouse model could provide important insight into human-specific/human-dominant metabolic pathways.

## 3. Materials and Methods

### 3.1. Chemicals and Regents

Atorvastatin (IUPAC: (3R,5R)-7-[2-(4-fluorophenyl)-5-isopropyl-3-phenyl-4-(phenylcarbamoyl)-1H-pyrrol-1-yl]-3,5-dihydroxyheptanoic acid), bosentan (IUPAC: 4-tert-butyl-N-[6-(2-hydroxyethoxy)-5-(2-methoxyphenoxy)[2,2′-bipyrimidin]-4-yl]benzenesulfonamide), and moxifloxacin (IUPAC: 1-cyclopropyl-6-fluoro-8-methoxy-7-[(4aS,7aS)-octahydro-6H-pyrrolo[3,4-b]pyridin-6-yl]-4-oxo-1,4-dihydro-3-quinolinecarboxylic acid) were obtained from TCI Europe (Zwijndrecht, Belgium). Epristeride (IUPAC: 17beta-(tert-butyl carbamoyl)androsta-3,5-diene-3-carboxylic acid) was obtained from Medchemexpress (Monmount Junction, NJ, USA). Cerivastatin (IUPAC: (3R,5S,6E)-7-[4-(4-fluorophenyl)-2,6-diisopropyl-5-(methoxymethyl)-3-pyridinyl]-3,5-dihydroxy-6-heptenoic acid), glipizide (IUPAC: N-(4-[N-(cyclohexylcarbamoyl)sulfamoyl]phenethyl)-5-methylpyrazine-2-carboxamide)), repaglinide (IUPAC: (S)-2-ethoxy-4-(2-((3-methyl-1-(2-(piperidin-1-yl) phenyl)butyl)amino)-2-oxoethyl)benzoic acid) and 2-hydroxypropyl-β-cyclodextrin (HP-β-CD)), pitavastatin (IUPAC: (3R,5S,6E)-7-[2-cyclopropyl-4-(4-fluorophenyl)-3-quinolinyl]-3,5-dihydroxy-6-heptenoic acid), telmisartan (IUPAC: 2-(4-{[4-methyl-6-(1-methyl-1H-1,3-benzodiazol-2-yl)-2-propyl-1H-1,3-benzodiazol-1-yl]methyl}phenyl)benzoic acid), and tesaglitazar (IUPAC: (2S)-2-ethoxy-3-[4-(2-{4-[(methylsulfonyl)oxy]phenyl}ethoxy)phenyl]propanoic acid) was obtained from SigmaAldrich (Schnelldorf, Germany). Albendazole (IUPAC: methyl [5-(propylthio)-1H-benzoimidazol-2-yl]carbamate), dextromethorphan (IUPAC: (4bS,8aR,9S)-3-methoxy-11-methyl-6,7,8,8a,9,10-hexahydro-5H-9,4b-(epiminoethano)phenanthrene), irbesartan (IUPAC: 2-butyl-3-{[2′-(1H-tetrazol-5-yl)[biphenyl]-4-yl]methyl}-1,3-diazaspiro[4.4]non-1-en-4-one), and PF-05089771 (IUPAC: 4-[2-(5-amino-1H-pyrazol-4-yl)-4-chlorophenoxy]-5-chloro-2-fluoro-N-(1,3-thiazol-4-yl)benzenesulfonamide) were obtained from Compound Management (AstraZeneca, Gothenburg, Sweden). HPLC-grade acetonitrile (ACN) was obtained from Fisher Scientific (Loughborough, UK); HPLC-grade formic acid (FA) was obtained from Pierce Biotechnology (Rockford, IL, USA). Other chemicals used included L-15 Leibovitz buffer with no phenol red and with L-glutamine (Gibco 21083-027, Billings, MT, USA), cryopreserved hepatocytes from BioIVT (West Sussex, UK), and Casyton 05 651 808 001 (Roche, Basel, Switzerland). Water was purified using a Milli-Q water purification system. All other chemicals were obtained from Compound Management (AstraZeneca, Gothenburg, Sweden) and were of analytical grade or the best equivalent.

### 3.2. Equipment

The equipment included a Tecan Freedom Evo robot, a Variomag Teleshake 70 plate shaker with TEC Control 485, Tecan 100 μL red tips, NUNC 1 mL 96-deepWell PP Plates (Natural; 260252, Thermo scientific, Waltham, MA, USA), conical NUNC plates (0.45 mL/well; 249944, Thermo Scientific), microwell lids (263339, Thermo Scientific), NUNC 96-well caps (natural; 276002, Thermo Scientific), Eppendorf pipettes, Ranin multi-pipettes, an Eppendorf Easypet, a Vacuset, a Grant Waterbath, a Sigma 4K15 Centrifuge, and a Casy Innovatis cell counter.

### 3.3. Hepatocyte Incubation

The hepatocytes were from ethically approved sources. Cryopreserved, pooled primary human hepatocytes (10 donors, male and female) and ICR/CD1 mouse hepatocytes (male) were obtained from BioIVT. L-15 Leibovitz buffer was placed in a water bath until it reached 37 °C. Cryopreserved cells were transferred from a −150 °C freezer on dry ice and immediately immersed in a preheated water bath kept at 37 °C. When only a small ice crystal could be seen, the contents were emptied into a 50 mL falcon tube filled with warm buffer (maximum 4 vials/falcon tube). The suspension was centrifuged at room temperature for 3 min at 50× *g*. The supernatant was removed, the pellet was re-suspended in a small volume of buffer, and the falcon tube was re-filled with buffer followed by another round of centrifugation. Thereafter, the supernatant was removed, and the pellet was dissolved in a small volume of buffer and diluted to a concentration of about 3–5 million cells/mL. After counting the cells using a Casy Cell counter, the suspension was diluted to 1 million cells/mL and kept at room temperature until further use. The viability cut-off was 80%.

A 245 µL aliquot of the hepatocyte suspension (1 million/mL) was added to a round-bottomed 96-deepwell plate using a manual multi-pipette. The deepwell plate was pre-incubated for 15 min at 37 °C and 13 Hz. Substrate solutions of the assay compounds were prepared in two plates using a robot (one plate for each species) as follows: 4 µL of a 10 mM DMSO stock solution for each plate, followed by the addition of 96 µL of 50% ACN and then the plate was mixed by shaking. From the two compound plates, 50 µL of each well was transferred and combined in a new plate, resulting in 100 µL per well and a dilution of 1:1.

The reaction was started by adding 5 µL of a 200 µM substrate solution, resulting in a final substrate concentration of 4 µM (0.04% DMSO, 1% ACN) and a cell concentration of 1 million cells/mL. The incubation was continued at 37 °C and 13 Hz. At each time point (0, 40, and 120 min), 50 µL of sample was taken out and quenched in 200 µL of cold ACN/MeOH (1:1) and the stopped plates were centrifuged for 20 min (at 4 °C and 4000× *g*). The supernatant was diluted by taking out 50 µL and mixing it with 100 µL of water. Albendazole and dextromethorphan were used as controls for the hepatocyte incubations.

### 3.4. Animals

Commercially available PXB-mice [20] were used for the current study. The animal study was conducted by Inotiv at the Department of Comparative Medicine, St. Louis University. The PXB-mouse^®^ used in the study is a chimeric mouse model with humanized livers, which was generated at PhoenixBio Co. Ltd, as described previously [47].

Three male PXB-mice (18–20 weeks old) with a human hepatocyte replacement index (measured by human albumin levels in whole blood) of at least 85% were provided by PhoenixBio Ltd (Hiroshima, Japan).

The animals were maintained under standard conditions (e.g., 12 h light/dark cycle with free access to food and water and controlled temperature and humidity conditions) for seven days to acclimatize them. Their body weights were normal for mice of this strain, age, and sex (17.3–20.2 g). Prior to dosing, the mice were acclimatized for an appropriate period prior to the start of the study (undertaken at Inotiv, 19 Worthington Access Drive, Maryland Heights, MO 63043, USA) in accordance with the Animal Welfare Act (9 CFR Parts 1, 2 and 3) and The Guide for the Care and Use of Laboratory Animals: Eighth Edition (Institute of Laboratory Animal Resources, National Academy Press, Washington, D.C., 2011). The protocol was reviewed and approved by the study facility’s Institutional Animal Care and Use Committee (IACUC) before the study commenced as per the IACUC’s standard operating procedures.

### 3.5. Animal Dosing

The treatment group was administered 1.5 mg/kg/total load of the cassette (total load: 0.5 mg/kg per test drug), formulated in 5% DMSO and 95% HP-β-CD [30% *w*/*v*] in sterile water for injection (solution) on Days 1 and 8 through IV bolus injection via the tail vein at a dose volume of 2 mL/kg. The mice were housed in metabolism cages immediately after dosing. Urine was collected at 0–4, 4–8, and 8–24 h post-dose. Serial blood samples (target: 10 μL ± 10% aliquots) taken from the tail vein were collected into heparin-coated microtubes at 5, 10, and 30 min and 1, 3, 8, and 24 h post-dose; the samples were centrifuged at 3200× *g* for 5 min at 4 °C and the blood was transferred into 1.5 mL cryovials. Blood samples were obtained from the control animals before the dose and at termination. The blood samples were kept frozen at − 80 °C.

### 3.6. Sample Preparation of In Vivo Samples from PXB-Mice for Metabolite Profiling

To obtain circulating metabolite profiles, individual whole blood samples from all time points were used (5 μL). The blood was supplemented with ACN (37.5 μL) and vortex-mixed to precipitate proteins, followed by centrifugation at 4,000 G for 20 min at 4 °C. The supernatants were diluted 1:1 with water. The samples from all three individuals were then pooled for each time point and injected into the LC–MS system. The urine from the mice was collected collectively and aliquots (50 μL) of urine at the 0–4, 4–8, and 8–12 h time points were mixed with 180 μL of ACN, vortexed, and centrifuged at 4,000 G for 20 min at 4 °C. The clear supernatant was diluted 1:1 with water and used for the LC–MS analysis.

### 3.7. UPLC–MS Method Used for Metabolite Profiling

Structural characterization of the metabolites and metabolite profiling was performed using either a Waters Acquity UPLC system fitted to a Waters Synapt G2-Si mass spectrometer (MS) or a ThermoFisher Vanquish U(H)PLC system coupled to a ThermoFisher Orbitrap Exploris 480 MS. The Waters Synapt G2-Si system, equipped with an electrospray ionization (ESI) source, was operated in positive-ion sensitivity mode over a mass range of *m*/*z* 50–1200 and the Orbitrap Exploris 480, equipped with an electrospray ionization (ESI) source, was operated in positive-ion and negative-ion modes over a mass range of *m*/*z* 120–1200. The reversed-phase gradient elution was performed on an Acquity U(H)PLC BEH C18 column (2.1 × 100 mm, 1.7 μm, Waters) at 45 °C. The mobile phases consisted of 0.1% FA in water (A) and ACN (B). The gradient used was as follows: 5% B at 0 min, increased to 40% B at 4.0 min, and increased to 95% at 6.5 min. The solvent composition was held at 95% B for 1.0 min and then decreased to 5% B at 7.5 min, followed by re-equilibration at 5% B for 0.5 min. For analysis, for each sample, 2 μL of the hepatocyte incubations, 5 μL of blood, or 10 μL of urine was injected. Full-scan MS spectra using the Waters Synapt G2-Si system were obtained using the MS^e^ mode over the mass range of 100–1200 Da in the low-energy function, and 50–1000 Da in the high-energy function. A scan time of 0.1 s was used, and centroid data were acquired. No trap-collision energy was used for the low-energy function, in the high-energy function, the trap-collision energy was ramped from 0 to 10 V. The cone voltage was set to 25 V for both the low- and high-energy functions. For dedicated MSMS, the *m*/*z* for the molecular ion was fixed in the quadrupole, and the mass range was set as 50–1000 Da. The trap-collision energy was ramped from 5 to 20 V. The transfer collision energy was set to 20 V. The cone voltage was set to 25 V. The tune parameters for all the MS analyses consisted of a capillary energy of 0.5 kV, sampling energy of 40 V, source temperature of 150 °C, desolvation temperature of 550 °C, cone gas flow of 0 L/h, desolvation gas flow of 1200 L/h, and nebulizer pressure of 6.5 Bar. Full-scan MS spectra using the Orbitrap Exploris 480 system were obtained over the mass range of 120–1200 Da, followed by a second AIF scan over the mass range of 100–1000 Da using normalized HCD collision energies of 20, 45, and 55 (%). The resolution of both scan events was set at 15,000, with polarity switching between the positive- and negative-ion modes. The spray voltage was set to 3500 V in the positive-ion mode and 2500 V in the negative-ion mode. The sheath gas and auxiliary gas flow was set to 50 and 10 (arbitrary units), respectively. The ion transfer tube temperature was 320 °C and the vaporizer temperature was 300 °C.

### 3.8. Metabolite Identification by MS

Metabolite identification was conducted by processing the generated data using the MetaboLynx™ XS browser (www.waters.com) or using MassMetaSite version 4.4.1 and the web-interphase ONIRO (Mass Analytica^TM^, https://mass-analytica.com/). A list of common Phase I and II transitions were used for the identification of the expected metabolites, and potential unexpected metabolites within the mass defect filtering region were also suggested by the software. Accurate masses of metabolites were determined from the protonated molecules in the positive ESI–TOF–MS mode or from the protonated and/or deprotonated molecules in the positive and negative ESI-Orbitrap-MS mode. The mass error for each proposed metabolite and fragment ion structure was <5 ppm (range: 0.1 to −1.4 mDa for metabolites seen in blood) compared to the theoretical exact mass. Drug-related materials below the cut-off (Synapt G2-Si absolute area ≥100 and ≥0.1% of total detected drug-related material; Orbitrap480 absolute area ≥4.2 × 10^4^ and ≥0.05% of total detected drug-related material) were labeled as not detected (ND). For those metabolites where no MSMS data could be acquired, identification was based on the accurate mass of the molecular ion and the fragmentation seen in the MS^e^ or AIF spectrum.

## 4. Conclusions

This study investigated the biotransformation of 12 different small-molecule drugs in PXB-mice (analysis of blood and urine) compared with hepatocytes from humans and mice and, when available, literature reports on human in vivo metabolism. The metabolites detected from these 12 drugs covered the majority of the Phase I and II transitions, such as hydroxylation, and N- and O-dealkylation and glucuronidation (Figure 1). For the investigated drugs, the metabolites detected in the blood and urine from the PXB-mice correlated well with the literature data of the metabolic patterns in humans in vivo (Table 2 and Table 3). It is worth noting that the human hepatocytes were able to detect most of the circulating metabolites (Table 2 and Table 3); hence, in the majority of cases, a good prediction of the metabolic pathways in humans will be obtained by investigating metabolite formation in human hepatocytes. In cases where the biotransformation pathways differ significantly between hepatocytes from human and nonclinical species, it could be useful to investigate metabolite formation in chimeric PXB-mice.

## Figures and Tables

**Table 1:** Metabolite profiling in PXB-mouse blood and urine, human and mouse hepatocytes, and in human plasma and urine.

Compound	Transformation	PXB blood	PXB urine	Human heps	Mouse heps	Human plasma [21]	Human urine
Atorvastatin	Parent	**+**	**+**	**+++**	**+**	**+++**	N/A
M1	4-Hydroxylation	**–**	**–**	**++**	**+**	**+**	N/A
M2	2-Hydroxylation	**+++**	**+**	**++**	**+**	**++**	N/A
M3	Lactone	**–**	**–**	**+**	**–**	**+**	N/A
M4	4-Hydroxylated Lactone	**–**	**–**	**+**	**–**	**+**	N/A
M5	2-Hydroxylated Lactone	**+++**	**+**	**+**	**+**	**+**	N/A
m6	β-Oxidation	**–**	**–**	**++**	**+++**	**–**	N/A
m7	β-Oxidation and 4-Hydroxylation	**–**	**–**	**+**	**–**	**–**	N/A
m8	β-Oxidation and 2-Hydroxylation	**+**	**–**	**+**	**+++**	**–**	N/A
m9	β-Oxidation and Desaturation and Hydroxylation	**–**	**–**	**–**	**+**	**–**	N/A
Compound	Transformation	PXB blood	PXB urine	Human heps	Mouse heps	Human plasma [23]	Human urine [23]
Bosentan	Parent	**+++**	**++**	**+++**	**+++**	**+++**	**+**
M1	Hydroxylation	**++**	**+++**	**+++**	**+++**	**+++**	**+++**
M2	Demethylation	**–**	**+**	**++**	**+**	**+**	**+**
M3	Hydroxylation and Demethylation	**–**	**+**	**+**	**+**	**–**	**++**
m4	2 x Oxidation and Dehydrogenation	**–**	**++**	**–**	**++**	**–**	**–**
m5	Oxidation and Glucuronidation	**–**	**–**	**–**	**+**	**–**	**–**
m6	Hydroxylation	**–**	**–**	**–**	**+**	**–**	**–**
	Other Metabolites	**–**	**–**	**–**	**–**	**+** **^ a^**	**+** **^ a^**
Compound	Transformation	PXB blood	PXB urine	Human heps	Mouse heps	Human plasma [24]	Human urine [24]
Cerivastatin	Parent	**+++**	**+**	**+++**	**++**	**+++**	**–**
M1	Demethylation	**++**	**+++**	**++**	**+**	**+**	**++**
M23	Hydroxylation	**++**	**+++**	**++**	**+**	**++**	**+++**
M24	Demethylation and Hydroxylation	**+**	**+++**	**+**	**+**	**–**	**+**
m27	Deoxygenation	**++**	**–**	**–**	**+++**	**–**	**–**
m28	Deoxygenation and Demethylation	**++**	**+**	**–**	**+++**	**–**	**–**
Compound	Transformation	PXB blood	PXB urine	Human heps	Mouse heps	Human plasma	Human urine
Epristeride	Parent	**+++**	**+++**	**+++**	**+++**	N/A	N/A
m1	Glucuronidation	**–**	**+++**	**++**	**+++**	N/A	N/A
m2	Hydroxylation	**–**	**–**	**+**	**–**	N/A	N/A
m3	Hydroxylation	**–**	**–**	**+**	**–**	N/A	N/A
Compound	Transformation	PXB blood	PXB urine	Human heps	Mouse heps	Human plasma [25]	Human urine [25]
Glipizide	Parent	**+++**	**+**	**+++**	**+++**	**+++**	**+**
M1	Hydroxylation	**+**	**+++**	**++**	**++**	**+**	**++**
M2	Hydroxylation	**+**	**++**	**+**	**+++**	**+**	**+**
M3	Hydroxylation	**+**	**++**	**+**	**++**	**–**	**++**
M4	Hydroxylation	**+**	**++**	**+**	**+++**	**–**	**+**
m5	Glucuronidation	**+**	**–**	**–**	**–**	**+**	**–**
m6	Dehydrogenation	**++**	**–**	**–**	**–**	**–**	**–**
m7	Hydroxylation	**–**	**+**	**–**	**+**	**–**	**–**
m8	Hydroxylation	**–**	**–**	**–**	**+**	**–**	**–**
m9	Hydroxylation	**–**	**–**	**–**	**+**	**–**	**–**
m10	Hydroxylation	**–**	**–**	**–**	**+**	**–**	**–**
m11	2 x Oxidation and Dehydrogenation	**–**	**–**	**–**	**+**	**–**	**–**
	Other Metabolites	**–**	**+**	**–**	**–**	**+** **^ a^**	**+** **^ a^**
Compound	Transformation	PXB blood	PXB urine	Human heps	Mouse heps	Human plasma [26]	Human urine [26]
Irbesartan	Parent	**+++**	**++**	**+++**	**+**	**+++**	**++**
M1	Cyclopentane Hydroxylation and Butyl hydroxylation	**–**	**+++**	**–**	**++**	**+**	**+++**
M2	Cyclopentane Hydroxylation and Butyl Carbonylation	**–**	**++**	**–**	**–**	**+**	**+++**
M3	Carboxylation	**–**	**++**	**–**	**+**	**+**	**+++**
M4	Butyl Hydroxylation	**+**	**++**	**+**	**+++**	**+**	**+++**
M5/M7	Cyclopentane Hydroxylation	**+**	**++**	**++**	**++**	**++**	**+++**
M6	Butyl Carbonylation	**+**	**+**	**–**	**+++**	**++**	**+++**
M8	N-Glucuronidation	**+**	**++**	**+++**	**– ^b^**	**++**	**++**
m9	N-Dealkylation and Butyl Hydroxylation	**++**	**++**	**–**	**+++**	**–**	**–** **^ c^**
m10	N-Dealkylation	**++**	**–**	**–**	**++**	**–**	**–** **^ c^**
m11	N-Dealkylation and Hydrolysis	**+**	**++**	**–**	**++**	**–**	**–** **^ c^**
Compound	Transformation	PXB blood	PXB urine	Human heps	Mouse heps	Human plasma [27]	Human urine [27]
Moxifloxacin	Parent	**+++**	**+++**	**+++**	**+++**	**+++**	**+++**
M1	N-Sulphation	**+**	**+**	**+**	**+**	**+**	**+**
M2	Glucuronidation	**++**	**++**	**+**	**++**	**++**	**++**
m3	Taurine Conjugation	**–**	**+**	**–**	**+**	**–**	**–**
Compound	Transformation	PXB blood	PXB urine	Human heps	Mouse heps	Human plasma	Human urine
PF-05089771	Parent	**+++**	**+++**	**+++**	**+++**	N/A	N/A
m1	N-Glucuronidation	**+**	**+**	**+**	**–**	N/A	N/A
m2	N-Glucuronidation	**+**	**+**	**+**	**–**	N/A	N/A
m3	N-Dealkylation	**++**	**+**	**+**	**+**	N/A	N/A
m4	Oxygenation	**+**	**++**	**++**	**++**	N/A	N/A
Compound	Transformation	PXB blood	PXB urine	Human heps	Mouse heps	Human plasma [28]	Human urine [28]
Pitavastatin	Parent	**+++**	**+++**	**+++**	**+**	**+++**	**++**
P. lactone	Lactone	**++**	**++**	**+++**	**–**	**+++**	**+++**
P. gluc.	Glucuronidation	**–**	**–**	**++**	**–**	**––**	**+++**
P. lactone gluc.	Lactone and Glucuronidation	**+**	**++**	**++**	**–**	**–**	**+++**
M2	Lactone and Dehydrogenation	**–**	**–**	**–**	**–**	**+**	**–**
M9	β-Oxidation	**–**	**–**	**–**	**–**	**–**	**+**
m10	β-Oxidation and Taurine Conjugation	**–**	**–**	**–**	**+++**	**–**	**–**
m11	β-Oxidation and Taurine Conjugation	**–**	**–**	**–**	**+++**	**–**	**–**
m13	Aromatic Hydroxylation	**–**	**++**	**–**	**++**	**–**	**–**
m14	Quinolone Dihydrodiol	**–**	**+++**	**++**	**-**	**–**	**–**
Compound	Transformation	PXB blood	PXB urine	Human heps	Mouse heps	Human plasma [29]	Human urine [29]
Repaglinide	Parent	**+++**	**++**	**+++**	**+**	**+++**	**+**
M1	Ring-Opening and Dealkylation	**–**	**–**	**+**	**+**	**++**	**+++**
M2	Ring-Opening and Carboxylation	**–**	**++**	**+++**	**+++**	**+++**	**+++**
M4	Piperidine Hydroxylation	**+**	**+++**	**+++**	**+**	**+**	**+**
M5	Deethylation	**–**	**–**	**+**	**++**	**+**	**+**
M7	Glucuronidation	**+**	**+++**	**+++**	**++**	**++**	**++**
m8	Piperidine Hydroxylation and Leucinol Hydroxylation	**+**	**+++**	**++**	**–**	**–**	**–** **^ d^**
m9	Leucinol Hydroxylation	**+**	**–**	**++**	**–**	**–**	**–** **^ d^**
m10	Piperidine Dehydrogenation	**++**	**–**	**+**	**+**	**–**	**–**
Secondary glucuron.	Oxidation and Glucuronidation	**–**	**+++**	**++**	**+++**	**–**	**–** **^ d^**
Compound	Transformation	PXB blood	PXB urine	Human heps	Mouse heps	Human plasma [30]	Human urine [30]
Telmisartan	Parent	**+++**	**+++**	**+++**	**+++**	**+++**	**+**
M1	Glucuronidation	**++**	**+++**	**++**	**+**	**+**	**+**
m2	Hydroxylation	**–**	**–**	**–**	**+**	**–**	**–**
m3	Hydroxylation	**++**	**++**	**–**	**–**	**–**	**–**
m4	N-dealkylation and Glucuronidation	**–**	**++**	**–**	**–**	**–**	**–**
Compound	Transformation	PXB blood	PXB urine	Human heps	Mouse heps	Human plasma [31]	Human urine [31]
Tesaglitazar	Parent	**+++**	**++**	**+++**	**+++**	**+++**	**++**
M1	Glucuronidation	**+**	**+**	**+**	**+**	**+**	**++**

+/++/+++ corresponds to low/medium/high levels of the detected compound (+ is colored amber whereas ++ and +++ are colored green); — (marked with red color) corresponds to not detected in PXB-mouse samples and hepatocytes and not reported for human plasma and urine; N/A—not applicable (no human in vivo literature data); M—metabolites found in humans in vivo; m—metabolites only detected in vitro or in vivo in animals. ^a^ Trace amounts of unidentified compounds. ^b^ M8 is not detected in the mouse hepatocytes, but glucuronide conjugates of M4 and M5/M7 are seen. ^c^ Detected unidentified polar compounds, suspected to be N-dealkylation compounds. ^d^ Major unidentified polar compounds detected.

**Table 2:** Human plasma metabolites detected in PXB-mouse blood, and in human and mouse hepatocytes.

Compound	PXB Blood	Human Heps	Mouse Heps
Atorvastatin	Green	Green	Amber *
Bosentan	Amber	Green	Green
Cerivastatin	Green	Green	Green
Epristeride	N/A	N/A	N/A
Glipizide	Green	Green	Green
Irbesartan	Amber	Red	Amber
Moxifloxacin	Green	Green	Green
PF-05089771	N/A	N/A	N/A
Pitavastatin	Green	Green	Red
Repaglinide	Red	Green	Green
Telmisartan	Green	Green	Green
Tesaglitazar	Green	Green	Green

Green: >75% of the human plasma metabolites were detected. Amber: 50–75% of the human plasma metabolites were detected. Red: <50% of the human plasma metabolites were detected. N/A: not applicable, i.e., no human plasma metabolites reported. * >75% of trace amounts of the human plasma metabolites was detected.

**Table 3:** Human urine metabolites detected in PXB-mouse blood, and in human and mouse hepatocytes.

Compound	PXB Blood	Human Heps	Mouse Heps
Atorvastatin	N/A	N/A	N/A
Bosentan	Green	Green	Green
Cerivastatin	Green	Green	Amber *
Epristeride	N/A	N/A	N/A
Glipizide	Green	Green	Green
Irbesartan	Green	Red	Amber
Moxifloxacin	Green	Green	Green
PF-05089771	N/A	N/A	N/A
Pitavastatin	Amber	Green	Red
Repaglinide	Amber	Green	Green
Telmisartan	Green	Green	Green
Tesaglitazar	Green	Green	Green

Green: >75% of the human plasma metabolites were detected. Amber: 50–75% of the human plasma metabolites were detected. Red: <50% of the human plasma metabolites were detected. N/A: Not applicable, i.e., no human plasma metabolites reported. * >75% of trace amounts of the human plasma metabolites was detected.

## Data Availability

The data presented in this study are available on request from the corresponding authors.

## Supplementary Materials

The supporting information can be downloaded at: https://www.mdpi.com/article/10.3390/ijms26031141/s1.

## References

[1] Martignoni M., Groothuis G.M.M., de Kanter R. (2006). Species differences between mouse, rat, dog, monkey and human CYP-mediated drug metabolism, inhibition and induction. Expert Opin. Drug Metab. Toxicol..

[2] Anderson S., Luffer-Atlas D., Knadler M.P. (2009). Predicting circulating human metabolites: How good are we?. Chem. Res. Toxicol..

[3] Leclercq L., Cuyckens F., Mannens G.S., de Vries R., Timmerman P., Evans D.C. (2009). Which human metabolites have we MIST? Retrospective analysis, practical aspects, and perspectives for metabolite identification and quantification in pharmaceutical development. Chem. Res. Toxicol..

[4] Walker D., Brady J., Dalvie D., Davis J., Dowty M., Duncan J.N., Nedderman A., Obach R.S., Wright P. (2009). A holistic strategy for characterizing the safety of metabolites through drug discovery and development. Chem. Res. Toxicol..

[5] Guengerich F.P., MacDonald J.S. (2007). Applying mechanisms of chemical toxicity to predict drug safety. Chem. Res. Toxicol..

[6] Smith D.A., Obach R.S. (2009). Metabolites in safety testing (MIST): Considerations of mechanisms of toxicity with dose, abundance, and duration of treatment. Chem. Res. Toxicol..

[7] Hasegawa M., Kawai K., Mitsui T., Taniguchi K., Monnai M., Wakui M., Ito M., Suematsu M., Peltz G., Nakamura M. (2011). The reconstituted ‘humanized liver’ in TK-NOG mice is mature and functional. Biochem. Biophys. Res. Commun..

[8] Nishimura T., Hu Y., Wu M., Pham E., Suemizu H., Elazar M., Liu M., Idilman R., Yurdaydin C., Angus P. (2013). Using chimeric mice with humanized livers to predict human drug metabolism and a drug-drug interaction. J. Pharmacol. Exp. Ther..

[9] de Jong Y.P., Rice C.M., Ploss A. (2010). New horizons for studying human hepatotropic infections. J. Clin. Investig..

[10] Katoh M., Yokoi T. (2007). Application of chimeric mice with humanized liver for predictive ADME. Drug Metab. Rev..

[11] Okumura H., Katoh M., Sawada T., Nakajima M., Soeno Y., Yabuuchi H., Ikeda T., Tateno C., Yoshizato K., Yokoi T. (2007). Humanization of excretory pathway in chimeric mice with humanized liver. Toxicol. Sci..

[12] Inoue T., Nitta K., Sugihara K., Horie T., Kitamura S., Ohta S. (2008). CYP2C9-catalyzed metabolism of S-warfarin to 7-hydroxywarfarin in vivo and in vitro in chimeric mice with humanized liver. Drug Metab. Dispos..

[13] Inoue T., Sugihara K., Ohshita H., Horie T., Kitamura S., Ohta S. (2009). Prediction of human disposition toward S-3H-warfarin using chimeric mice with humanized liver. Drug Metab. Pharmacokinet..

[14] Yamazaki H., Kuribayashi S., Inoue T., Tateno C., Nishikura Y., Oofusa K., Harada D., Naito S., Horie T., Ohta S. (2010). Approach for In Vivo Protein Binding of 5-n-Butyl-pyrazolo[1,5-a]pyrimidine Bioactivated in Chimeric Mice with Humanized Liver by Two-Dimensional Electrophoresis with Accelerator Mass Spectrometry. Chem. Res. Toxicol..

[15] Kitamura S., Nitta K., Tayama Y., Tanoue C., Sugihara K., Inoue T., Horie T., Ohta S. (2008). Aldehyde oxidase-catalyzed metabolism of N1-methylnicotinamide in vivo and in vitro in chimeric mice with humanized liver. Drug Metab. Dispos..

[16] Sugahara G., Ishida Y., Sun J., Tateno C., Saito T. (2020). Art of Making Artificial Liver: Depicting Human Liver Biology and Diseases in Mice. Semin. Liver Dis..

[17] Tateno C., Kawase Y., Tobita Y., Hamamura S., Ohshita H., Yokomichi H., Sanada H., Kakuni M., Shiota A., Kojima Y. (2015). Generation of Novel Chimeric Mice with Humanized Livers by Using Hemizygous cDNA-uPA/SCID Mice. PLoS ONE.

[18] Nishimura M., Yoshitsugu H., Yokoi T., Tateno C., Kataoka M., Horie T., Yoshizato K., Naito S. (2005). Evaluation of mRNA expression of human drug-metabolizing enzymes and transporters in chimeric mouse with humanized liver. Xenobiotica.

[19] Kakuni M., Yamasaki C., Tachibana A., Yoshizane Y., Ishida Y., Tateno C. (2014). Chimeric Mice with Humanized Livers: A Unique Tool for in Vivo and in Vitro Enzyme Induction Studies. Int. J. Mol. Sci..

[20] PhoenixBio PXB-mouse. https://www.phoenixbio.com/products/pxb-mouse.

[21] Stangier J., Schmid J., Turck D., Switek H., Verhagen A., Peeters P.A., van Marle S.P., Tamminga W.J., Sollie F.A., Jonkman J.H. (2000). Absorption, metabolism, and excretion of intravenously and orally administered [14C]telmisartan in healthy volunteers. J. Clin. Pharmacol..

[22] Black A.E., Sinz M.W., Hayes R.N., Woolf T.F. (1998). Metabolism and excretion studies in mouse after single and multiple oral doses of the 3-hydroxy-3-methylglutaryl-CoA reductase inhibitor atorvastatin. Drug Metab. Dispos..

[23] Lennernas H. (2003). Clinical pharmacokinetics of atorvastatin. Clin. Pharmacokinet..

[24] Weber C., Gasser R., Hopfgartner G. (1999). Absorption, excretion, and metabolism of the endothelin receptor antagonist bosentan in healthy male subjects. Drug Metab. Dispos..

[25] Muck W. (2000). Clinical pharmacokinetics of cerivastatin. Clin. Pharmacokinet..

[26] Tan B., Yang A., Yuan W., Li Y., Jiang L., Jiang J., Qiu F. (2017). Simultaneous determination of glipizide and its four hydroxylated metabolites in human urine using LC-MS/MS and its application in urinary phenotype study. J. Pharm. Biomed. Anal..

[27] Chando T.J., Everett D.W., Kahle A.D., Starrett A.M., Vachharajani N., Shyu W.C., Kripalani K.J., Barbhaiya R.H. (1998). Biotransformation of irbesartan in man. Drug Metab. Dispos..

[28] Stass H., Kubitza D. (1999). Pharmacokinetics and elimination of moxifloxacin after oral and intravenous administration in man. J. Antimicrob. Chemother..

[29] Fujino H., Kojima J., Yamada Y., Kanda H., Kimata H. (1999). Studies on the Metabolic Fate of NK-104, a New Inhibitor of HMG-CoA Reductase (4): Interspecies Variation in the Laboratory Animals and Humans. Xenobio. Metabol. Dispos..

[30] van Heiningen P.N., Hatorp V., Kramer Nielsen K., Hansen K.T., van Lier J.J., De Merbel N.C., Oosterhuis B., Jonkman J.H. (1999). Absorption, metabolism and excretion of a single oral dose of (14)C-repaglinide during repaglinide multiple dosing. Eur. J. Clin. Pharmacol..

[31] Ericsson H., Hamren B., Bergstrand S., Elebring M., Fryklund L., Heijer M., Ohman K.P. (2004). Pharmacokinetics and metabolism of tesaglitazar, a novel dual-acting peroxisome proliferator-activated receptor alpha/gamma agonist, after a single oral and intravenous dose in humans. Drug Metab. Dispos..

[32] Dingemanse J., van Giersbergen P.L. (2004). Clinical pharmacology of bosentan, a dual endothelin receptor antagonist. Clin. Pharmacokinet..

[33] Boberg M., Angerbauer R., Kanhai W.K., Karl W., Kern A., Radtke M., Steinke W. (1998). Biotransformation of cerivastatin in mice, rats, and dogs in vivo. Drug Metab. Dispos..

[34] Kaspera R., Naraharisetti S.B., Tamraz B., Sahele T., Cheesman M.J., Kwok P.Y., Marciante K., Heckbert S.R., Psaty B.M., Totah R.A. (2010). Cerivastatin in vitro metabolism by CYP2C8 variants found in patients experiencing rhabdomyolysis. Pharmacogenet Genom..

[35] Boberg M., Angerbauer R., Fey P., Kanhai W.K., Karl W., Kern A., Ploschke J., Radtke M. (1997). Metabolism of cerivastatin by human liver microsomes in vitro. Characterization of primary metabolic pathways and of cytochrome P450 isozymes involved. Drug Metab. Dispos..

[36] Schmidt H.A., Schoog M., Schweer K.H., Winkler E. (1973). Pharmacokinetics and pharmacodynamics as well as metabolism following orally and intravenously administered C14-glipizide, a new antidiabetic. Diabetologia.

[37] Balant L., Zahnd G., Gorgia A., Schwarz R., Fabre J. (1973). Pharmacokinetics of glipizide in man: Influence of renal insufficiency. Diabetologia.

[38] Fuccella L.M., Tamassia V., Valzelli G. (1973). Metabolism and kinetics of the hypoglycemic agent glipizide in man--comparison with glibenclamide. J. Clin. Pharmacol. New Drugs.

[39] Chiu S.H., Huskey S.W. (1998). Species differences in N-glucuronidation. Drug Metab. Dispos..

[40] Kaivosaari S., Finel M., Koskinen M. (2011). N-glucuronidation of drugs and other xenobiotics by human and animal UDP-glucuronosyltransferases. Xenobiotica.

[41] Fujino H., Yamada I., Shimada S., Yoneda M., Kojima J. (2003). Metabolic fate of pitavastatin, a new inhibitor of HMG-CoA reductase: Human UDP-glucuronosyltransferase enzymes involved in lactonization. Xenobiotica.

[42] Yamada I., Fujino H., Shimada S., Kojima J. (2003). Metabolic fate of pitavastatin, a new inhibitor of HMG-CoA reductase: Similarities and difference in the metabolism of pitavastatin in monkeys and humans. Xenobiotica.

[43] Bidstrup T.B., Bjornsdottir I., Sidelmann U.G., Thomsen M.S., Hansen K.T. (2003). CYP2C8 and CYP3A4 are the principal enzymes involved in the human in vitro biotransformation of the insulin secretagogue repaglinide. Br. J. Clin. Pharmacol..

[44] Gan J., Chen W., Shen H., Gao L., Hong Y., Tian Y., Li W., Zhang Y., Tang Y., Zhang H. (2010). Repaglinide-gemfibrozil drug interaction: Inhibition of repaglinide glucuronidation as a potential additional contributing mechanism. Br. J. Clin. Pharmacol..

[45] Bauer E., Beschke K., Ebner T., Greischel A., Heinle R., Prox A. (1997). Biotransformation of [14C] repaglinide in human, cynomolgus monkey, dog, rabbit, rat and mouse (abstract 1282). Diabetologia.

[46] Chen A.A., Thomas D.K., Ong L.L., Schwartz R.E., Golub T.R., Bhatia S.N. (2011). Humanized mice with ectopic artificial liver tissues. Proc. Natl. Acad. Sci. USA.

[47] Tateno C., Yoshizane Y., Saito N., Kataoka M., Utoh R., Yamasaki C., Tachibana A., Soeno Y., Asahina K., Hino H. (2004). Near completely humanized liver in mice shows human-type metabolic responses to drugs. Am. J. Pathol..

[48] Samuelsson K., Pickup K., Sarda S., Swales J.G., Morikawa Y., Schulz-Utermoehl T., Hutchison M., Wilson I.D. (2012). Pharmacokinetics and metabolism of midazolam in chimeric mice with humanised livers. Xenobiotica.

[49] Serres M.D., Bowers G., Boyle G., Beaumont C., Castellino S., Sigafoos J., Dave M., Roberts A., Shah V., Olson K. (2011). Evaluation of a chimeric (uPA+/+)/SCID mouse model with a humanized liver for prediction of human metabolism. Xenobiotica.

[50] Alcock S. (1970). An anti-inflammatory compound: Non-toxic to animals but with an adverse action in man. Proc. Eur. Soc. Study Drug Toxic..

[51] Hart F.D., Bain L.S., Huskisson E.C., Littler T.R., Taylor R.T. (1970). Hepatic effects of fenclozic acid. Ann. Rheum. Dis..

[52] Ekdahl A., Weidolf L., Baginski M., Morikawa Y., Thompson R.A., Wilson I.D. (2018). The metabolic fate of fenclozic acid in chimeric mice with a humanized liver. Arch. Toxicol..

