# Supplementary material for: Investigation of Biotransformation Pathways in a Chimeric Mouse with a Humanized Liver
Source: Int J Mol Sci. 2025 Jan 28;26(3):1141. doi: 10.3390/ijms26031141 (PMC11818726; doi:10.3390/ijms26031141)
Supplement: Supplementary file 1 [file ijms-26-01141-s001.zip › ijms-3412284-supplementary.pdf]

Supplementary Materials

Investigation of Biotransformation Pathways in a Chimeric Mouse with a Humanized Liver

Isabella B. Karlsson [1,*], Anja Ekdahl [1], Hugh Etchingham-Coll [1], Xue-Qing Li [1], Cecilia Ericsson [1], Marie Ahlqvist [1], Kristin Samuelsson [1,*]

Drug Metabolism and Pharmacokinetics, Research and Early Development, Cardiovascular, Renal and Metabolism, BioPharmaceuticals R&D, AstraZeneca, Gothenburg, Sweden

* Correspondence: isabella.bonnerkarlsson@astrazeneca.com (I.K.); kristin.samuelsson1@astrazeneca.com (K.S.)

Contents

S1. Workflow 3
Figure S1. Workflow 3
S2. Metabolite Profiling of Atorvastatin 4
Figure S2. Metabolic Pathways of Atorvastatin. 4
S3. Metabolite Profiling of Bosentan 5
Figure S3. Metabolic Pathways of Bosentan. 5
S4. Metabolite Profiling of Cerivastatin 6
Figure S4. Metabolic pathway of Cerivastatin. 6
S5. Metabolite Profiling of Epristeride 7
Figure S5. Metabolic Pathways of Epristeride. 7
S6. Metabolite Profiling of Glipizide 8
Figure S6. Metabolic Pathways of Glipizide. 8
S7. Metabolite Profiling of Irbesartan 9
Figure S7. Metabolic Pathways of Irbesartan. 9
S8. Metabolite Profiling of Moxifloxacin 10
Figure S8. Metabolic Pathways of Moxifloxacin. 10
S9. Metabolite Profiling of PF-05089771 11
Figure S9. Metabolic Pathways of PF-05089771. 11
S10. Metabolite Profiling of Pitavastatin 12
Figure S10. Metabolic Pathways of Pitavastatin. 12
S11. Metabolite Profiling of Repaglinide 13
Figure S11. Metabolic Pathways of Repaglinide. 13
S12. Metabolite Profiling of Telmisartan 14
Figure S12. Metabolic Pathways of Telmisartan. 14
S13. Metabolite Profiling of Tesaglitazar 15
Figure S13. Metabolic Pathway of Tesaglitazar. 15
References 16

S1. Workflow

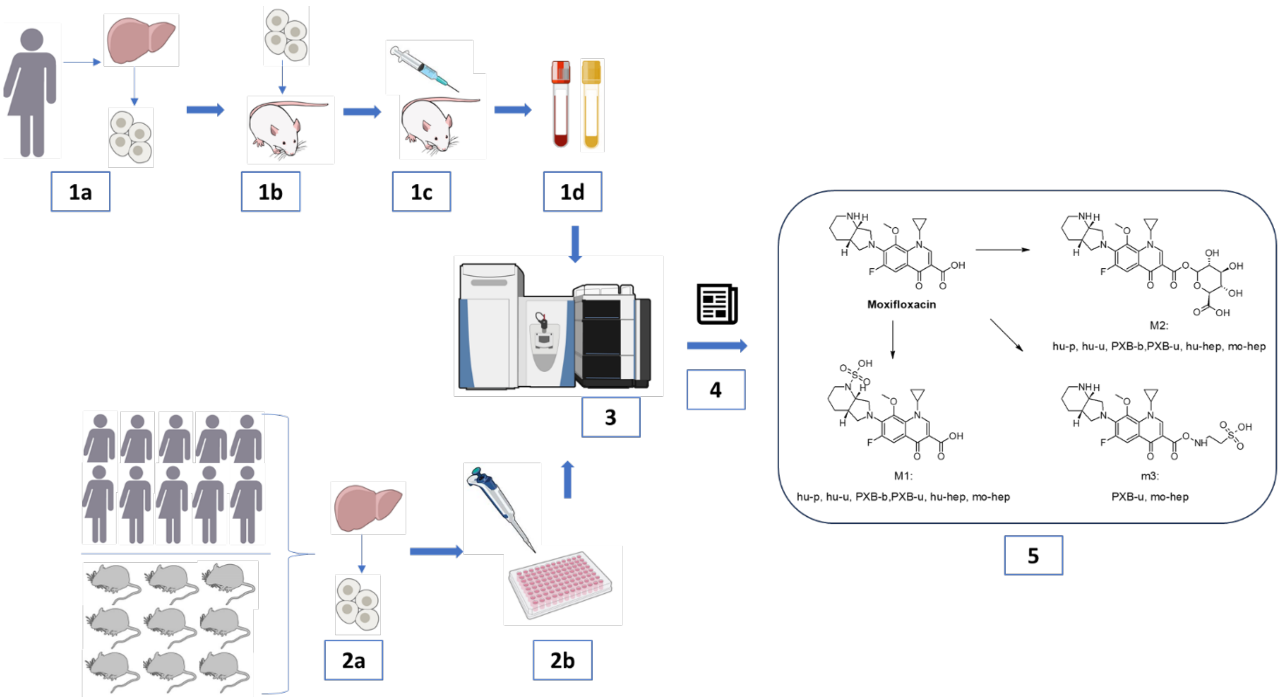

Figure S1. Workflow

Figure S1 shows a schematic presentation of the workflow used in the study. **1a-d** represent methodology for generation of biofluids from the PXB-mice as follows. **1a**) Hepatocytes are generated from a single human donor. **1b**) Human hepatocytes are transplanted into severe combined immunodeficient mice who have had their mouse hepatocytes ablated. The human hepatocytes will form a mature organ which express human specific Phase I and II enzymes. **1c**) The investigated compounds are administered via intravenous bolus tail vein injection. Cassette dosing with three compounds per cassette was employed (0.5 mg/kg/test compound). Each group contained three PXB-mice. **1d**) Blood was collected after 5 min, 10 min, 30 min, 1 h, 3 h, 8 h, and 24 h post-dose. Urine was collected at 0-4 h, 4-8 h, and 8-24 h. **2a-b** represent the hepatocyte incubations, as follows. **2a**) Human hepatocytes from a mixed gender pool of 10 donors and mouse hepatocytes from a pool of male CD-1 mice were used. **2b**) The hepatocyte incubation was performed using a substrate concentration of 4 µM and 1 million cells/mL. Samples were taken after 0, 40 and 120 min. **3**) Metabolite profiling and structural characterization was performed using U(H)PLC-HRMS. **4**) Literature data on human *in vivo* metabolites detected in plasma/blood and urine were compiled. **5**) Metabolite profiles were prepared for each compound comparing the metabolic pathways observed in PXB-mice with human and mouse hepatocytes and human *in vivo* literature data. *Illustrations from NIAID NIH BIOART Source (bioart.niaid.nih.gov/bioart) has been used to create the figure.*

S2. Metabolite Profiling of Atorvastatin

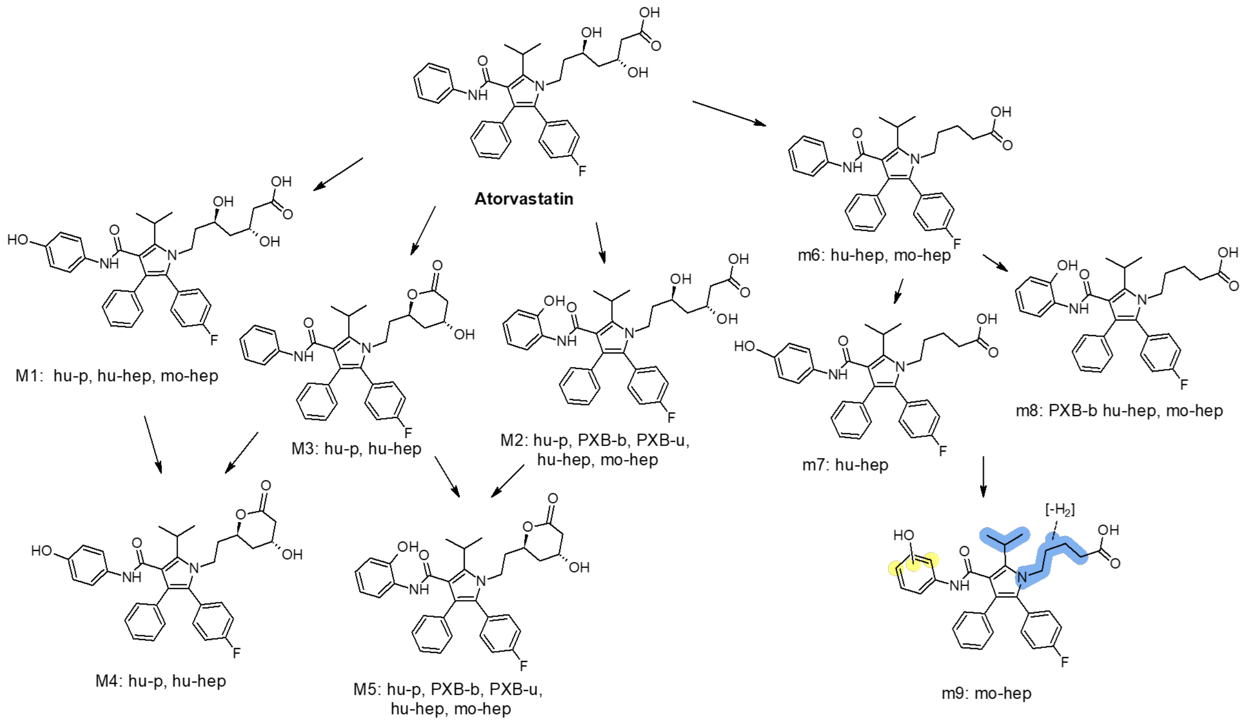

Figure S2. Metabolic Pathways of Atorvastatin.

Human plasma, hu-p; PXB-mouse blood, PXB-b; PXB-mouse urine, PXB-u; human hepatocytes, h-hep; mouse hepatocytes: mo-hep. Information on human *in vivo* metabolites in circulation is from Lennernas [1].

S3. Metabolite Profiling of Bosentan

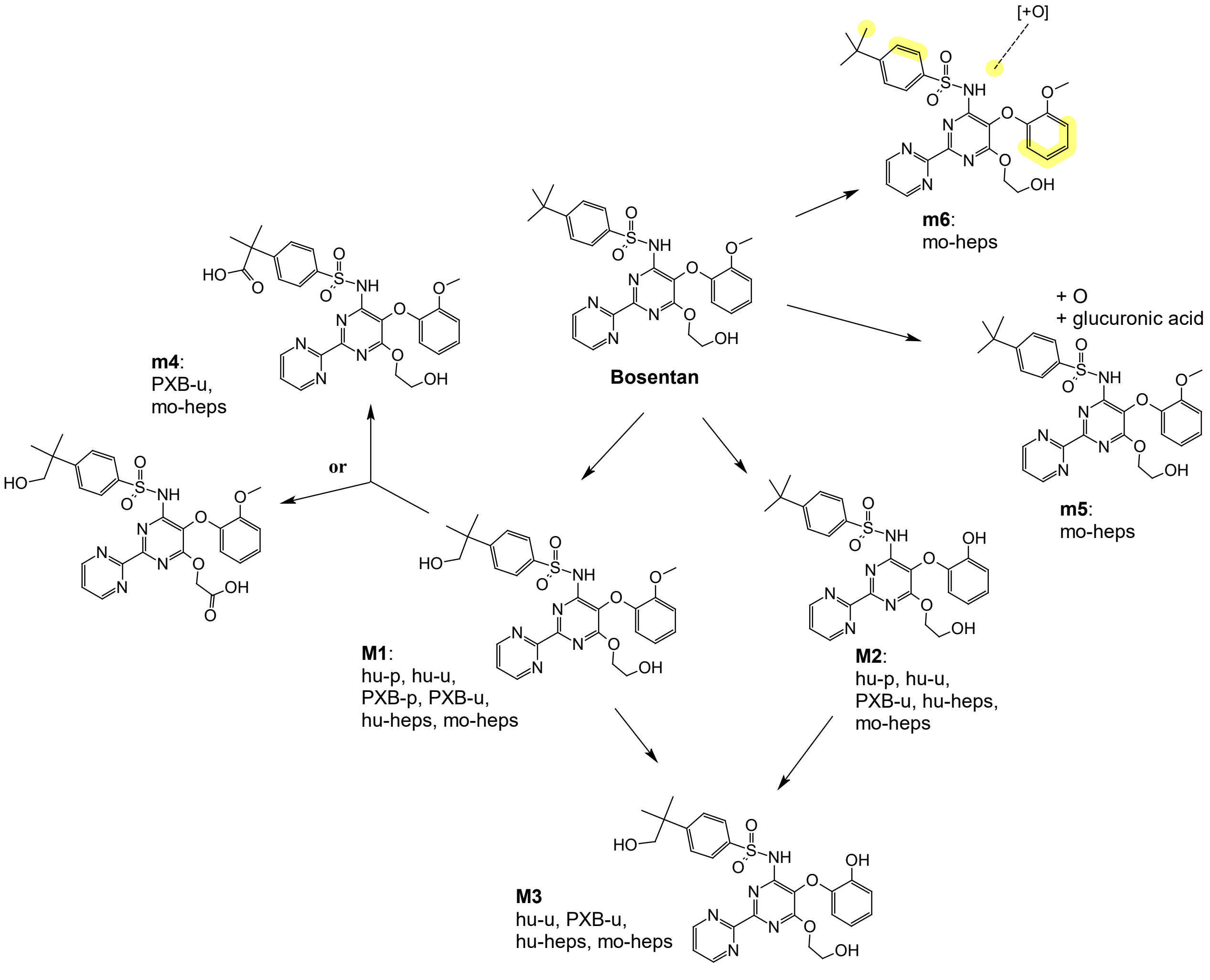

Figure S3. Metabolic Pathways of Bosentan.

Human plasma: hu-p, human urine: h-u, PXB-mouse® blood: PXB-b, PXB-mouse® urine: PXB-u, human hepatocytes: h-hep, mouse hepatocytes: mo-hep. Information on human *in vivo* metabolites in circulation and in urine is from Weber et al. [2]

S4. Metabolite Profiling of Cerivastatin

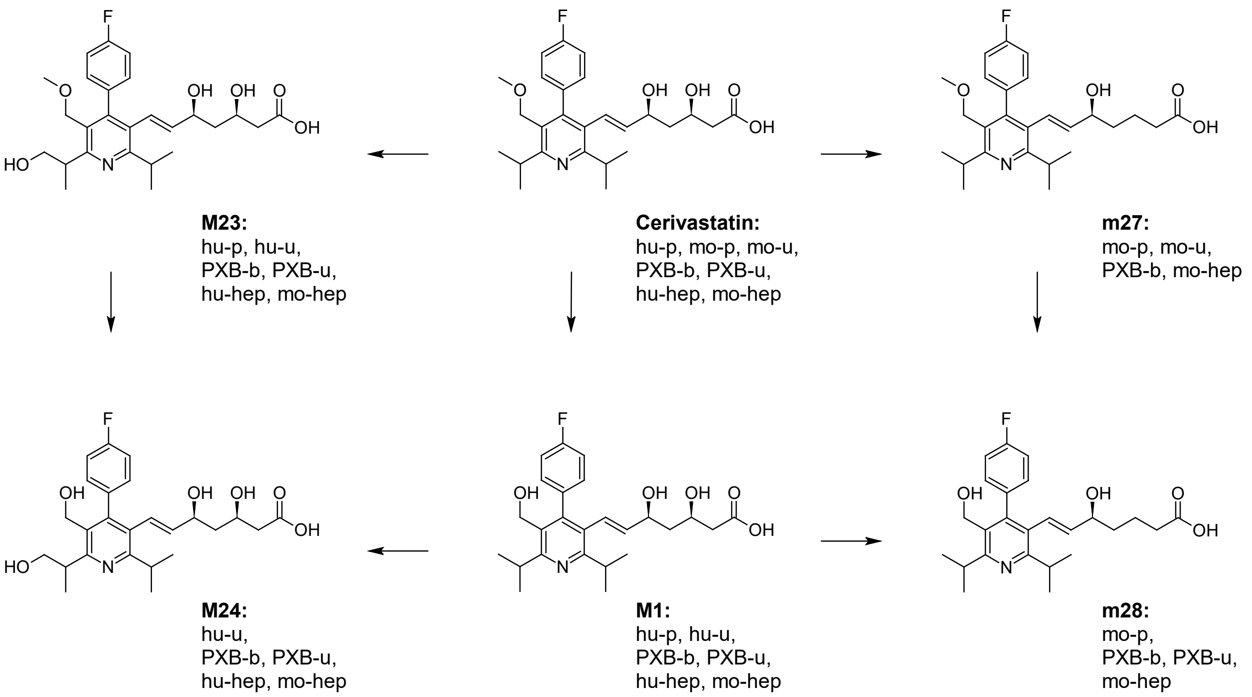

Figure S4. Metabolic pathway of Cerivastatin.

Human plasma: hu-p, human urine: mouse plasma: mo-p, mouse urine: mo-u, PXB-mouse® blood: PXB-b, PXB-mouse® urine: PXB-u, human hepatocytes: h-hep, mouse hepatocytes: mo-hep. Information on human *in vivo* metabolites in circulation and in urine is from Muck [3].

S5. Metabolite Profiling of Epristeride

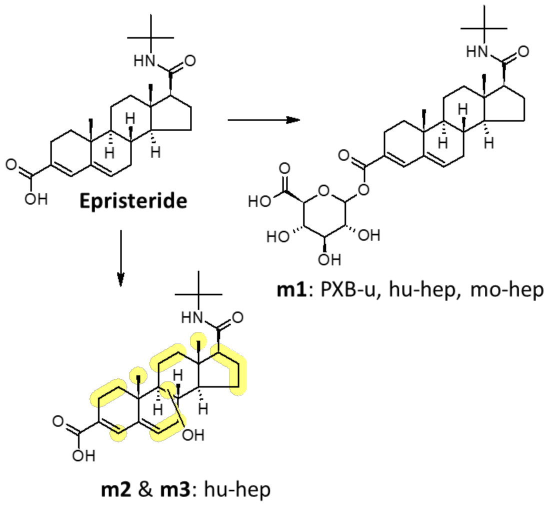

Figure S5. Metabolic Pathways of Epristeride.

PXB-mouse urine, PXB-u; human hepatocytes, h-hep; mouse hepatocytes: mo-hep.

S6. Metabolite Profiling of Glipizide

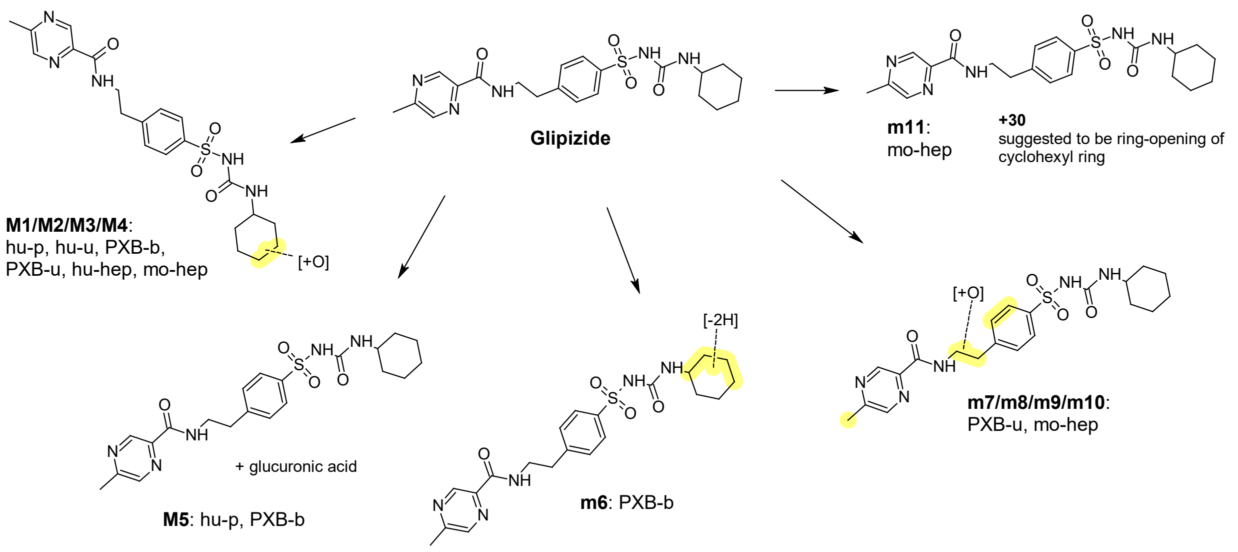

Figure S6. Metabolic Pathways of Glipizide.

Human plasma: hu-p, human urine: h-u, PXB-mouse® blood: PXB-b, PXB-mouse® urine: PXB-u, human hepatocytes: h-hep, mouse hepatocytes: mo-hep. Information on human *in vivo* metabolites in circulation and in urine is from Tan et al. [4]

S7. Metabolite Profiling of Irbesartan

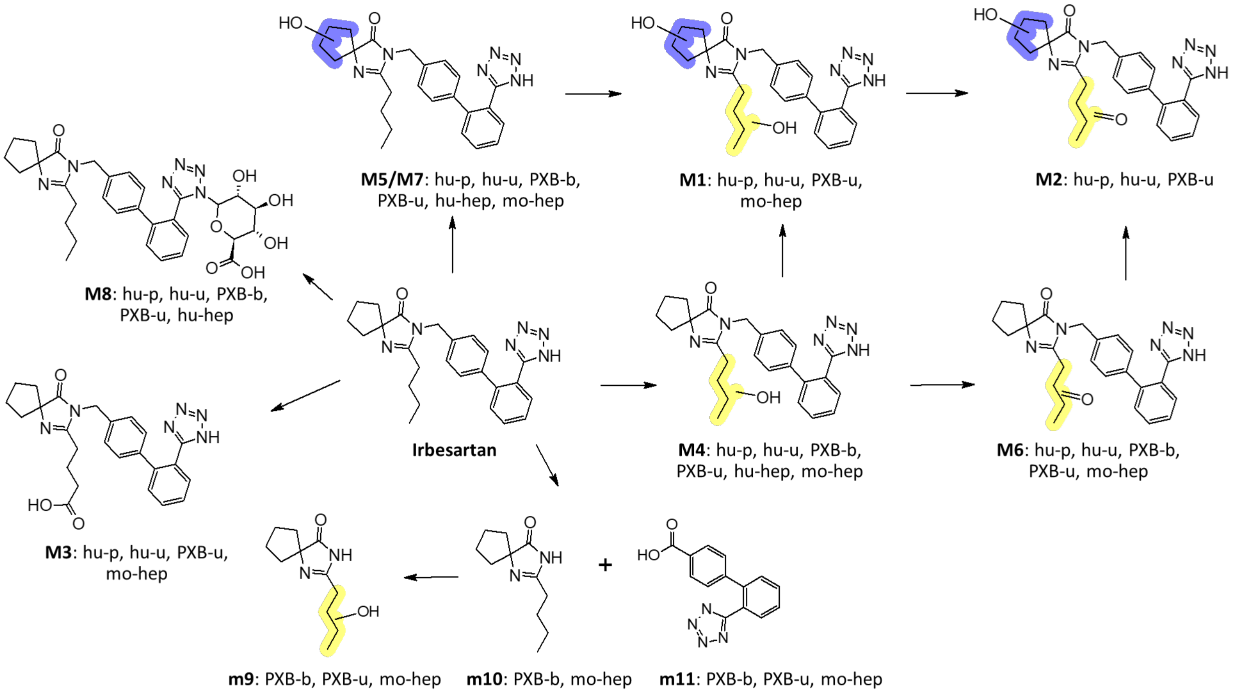

Figure S7. Metabolic Pathways of Irbesartan.

Human plasma: hu-p, human urine: h-u, PXB-mouse® blood: PXB-b, PXB-mouse® urine: PXB-u, human hepatocytes: h-hep, mouse hepatocytes: mo-hep. Information on human *in vivo* metabolites in circulation and in urine is from Chando et al. [5]

S8. Metabolite Profiling of Moxifloxacin

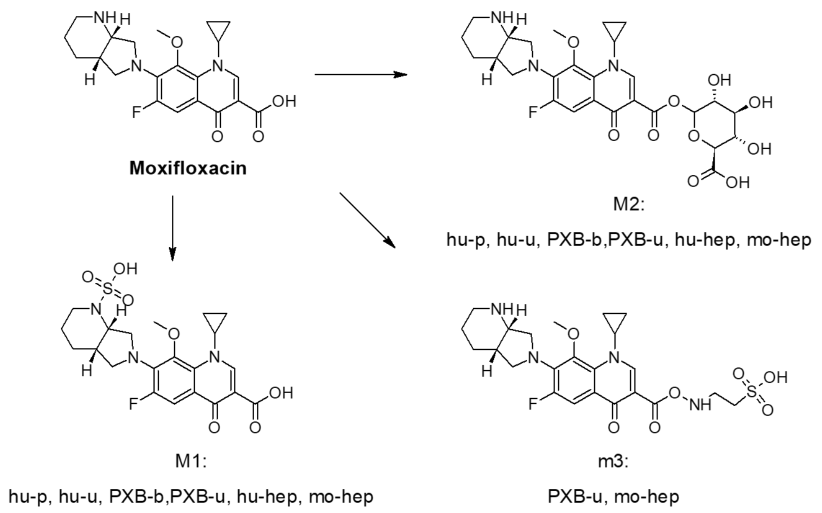

Figure S8. Metabolic Pathways of Moxifloxacin.

Human plasma, hu-p; human urine, h-u; PXB-mouse blood, PXB-b; PXB-mouse urine, PXB-u; human hepatocytes, h-hep; mouse hepatocytes: mo-hep. Information on human *in vivo* metabolites in circulation and in urine is from Stass and Kubitza [6].

S9. Metabolite Profiling of PF-05089771

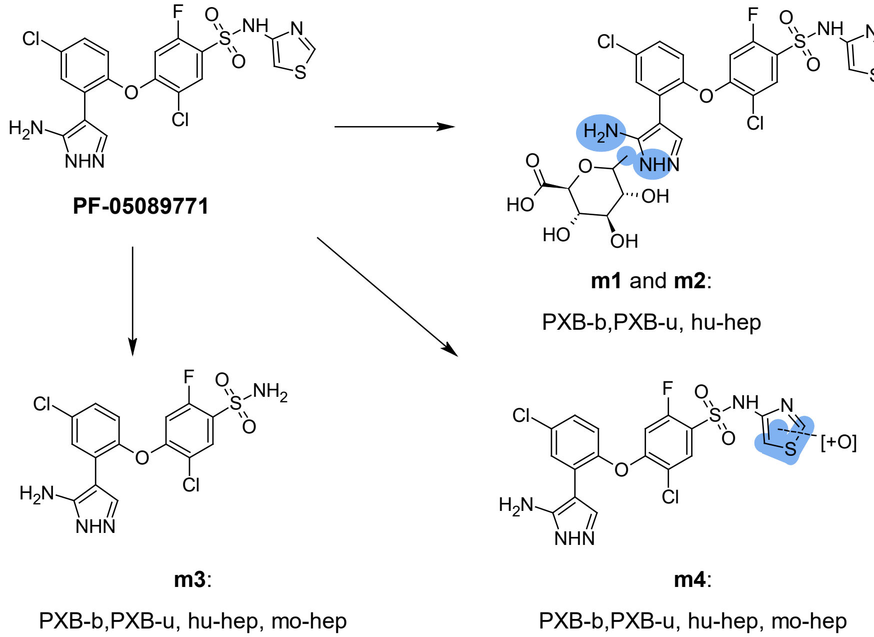

Figure S9. Metabolic Pathways of PF-05089771.

PXB-mouse blood, PXB-b; PXB-mouse urine, PXB-u; human hepatocytes, h-hep; mouse hepatocytes: mo-hep.

S10. Metabolite Profiling of Pitavastatin

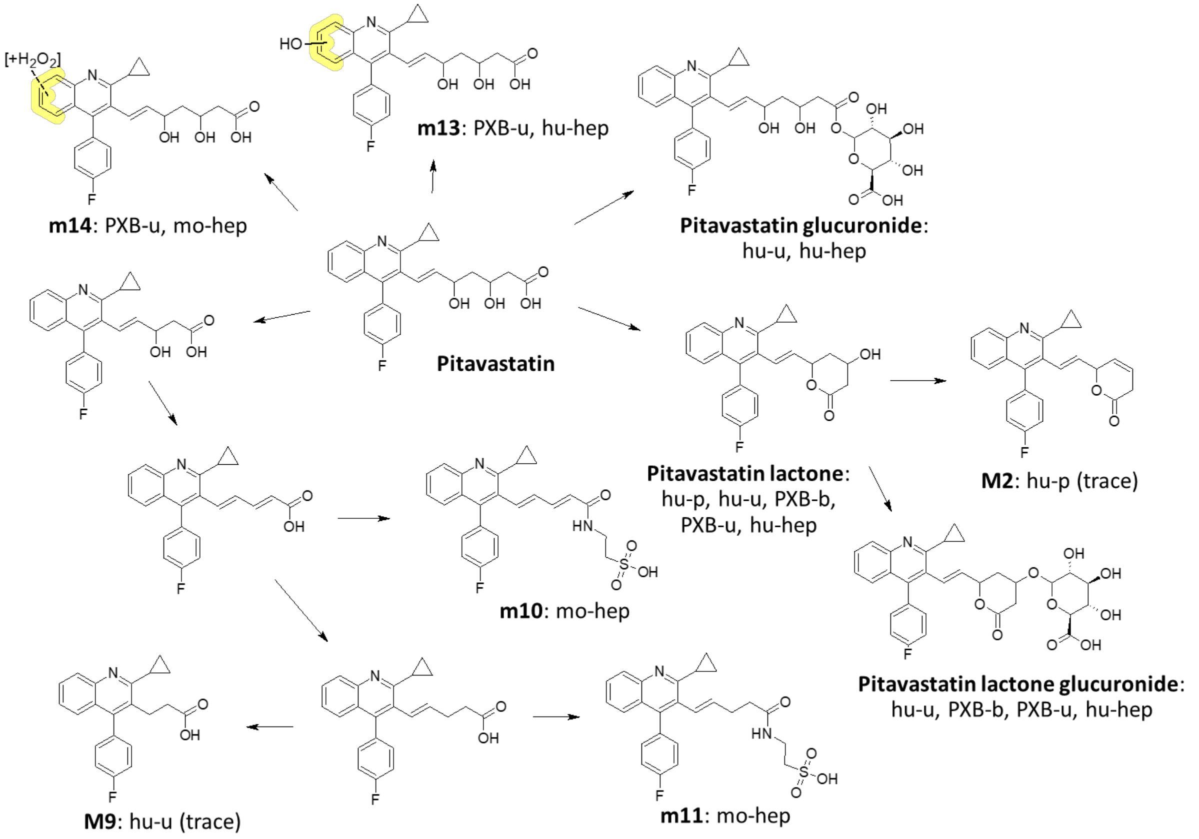

Figure S10. Metabolic Pathways of Pitavastatin.

Human plasma: hu-p, human urine: h-u, PXB-mouse® blood: PXB-b, PXB-mouse® urine: PXB-u, human hepatocytes: h-hep, mouse hepatocytes: mo-hep. Information on human *in vivo* metabolites in circulation and in urine is from Fujino et al. [7]

S11. Metabolite Profiling of Repaglinide

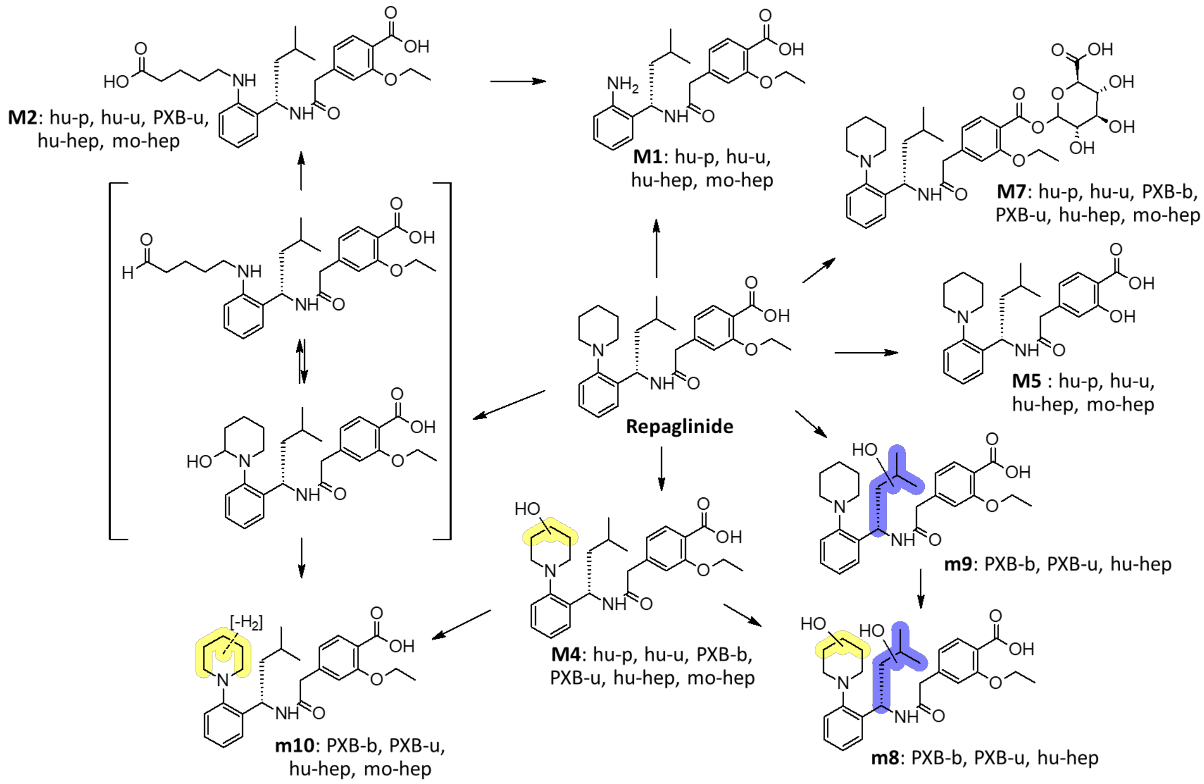

Figure S11. Metabolic Pathways of Repaglinide.

Human plasma: hu-p, human urine: h-u, PXB-mouse® blood: PXB-b, PXB-mouse® urine: PXB-u, human hepatocytes: h-hep, mouse hepatocytes: mo-hep. Information on human *in vivo* metabolites in circulation and in urine is from van Heiningen et al. [8]

S12. Metabolite Profiling of Telmisartan

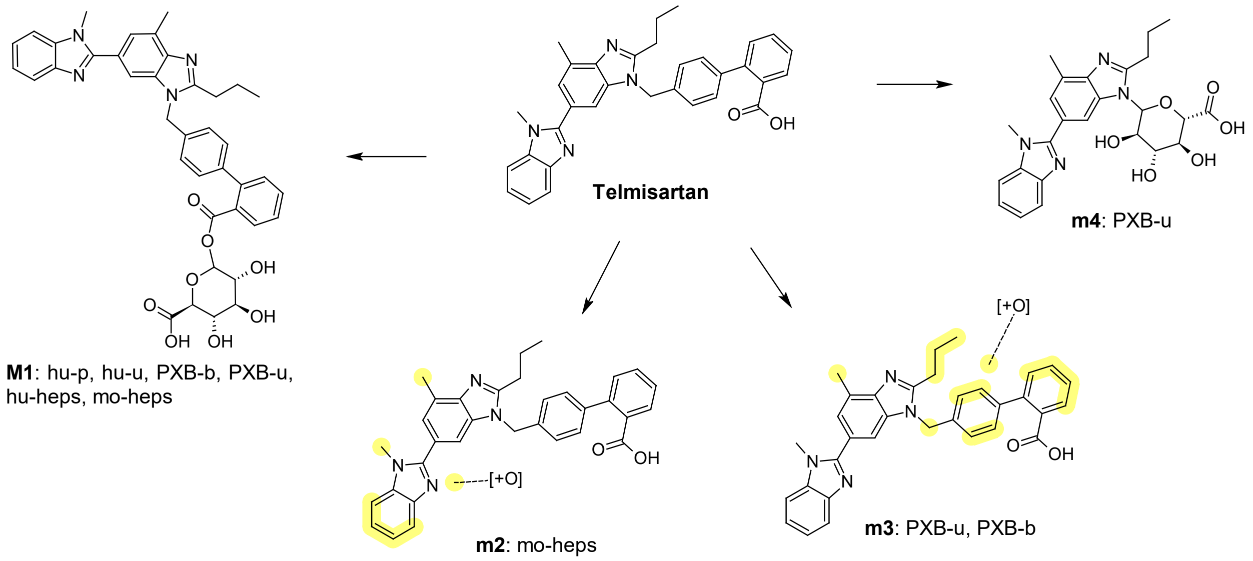

Figure S12. Metabolic Pathways of Telmisartan.

Human plasma: hu-p, human urine: h-u, PXB-mouse® blood: PXB-b, PXB-mouse® urine: PXB-u, human hepatocytes: h-hep, mouse hepatocytes: mo-hep. Information on human *in vivo* metabolites in circulation and in urine is from Stangier at el. [9]

S13. Metabolite Profiling of Tesaglitazar

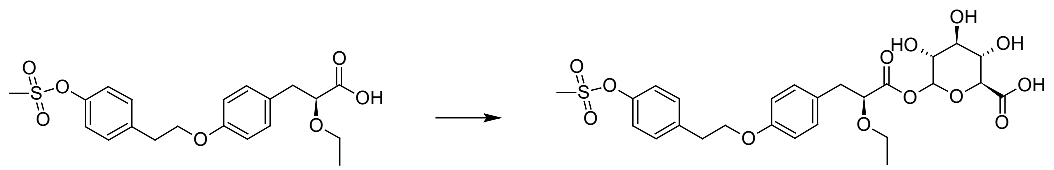

Tesaglitazar:
hu-p, hu-u,
PXB-b, PXB-u,
hu-hep, mo-hep

M1:
hu-p, hu-u,
PXB-b, PXB-u,
hu-hep, mo-hep

Figure S13. Metabolic Pathway of Tesaglitazar. Human plasma: hu-p, human urine: h-u, PXB-mouse® blood: PXB-b, PXB-mouse® urine: PXB-u, human hepatocytes: h-hep, mouse hepatocytes: mo-hep. Information on human *in vivo* metabolites in circulation and in urine is from Ericsson et al. [10]

References

1. Lennernas, H., Clinical pharmacokinetics of atorvastatin. *Clin Pharmacokinet* **2003,** 42, (13), 1141-60.
2. Weber, C.; Gasser, R.; Hopfgartner, G., Absorption, excretion, and metabolism of the endothelin receptor antagonist bosentan in healthy male subjects. *Drug Metab Dispos* **1999,** 27, (7), 810-5.
3. Muck, W., Clinical pharmacokinetics of cerivastatin. *Clin Pharmacokinet* **2000,** 39, (2), 99-116.
4. Tan, B.; Yang, A.; Yuan, W.; Li, Y.; Jiang, L.; Jiang, J.; Qiu, F., Simultaneous determination of glipizide and its four hydroxylated metabolites in human urine using LC-MS/MS and its application in urinary phenotype study. *J Pharm Biomed Anal* **2017,** 139, 179-186.
5. Chando, T. J.; Everett, D. W.; Kahle, A. D.; Starrett, A. M.; Vachharajani, N.; Shyu, W. C.; Kripalani, K. J.; Barbhaiya, R. H., Biotransformation of irbesartan in man. *Drug Metab Dispos* **1998,** 26, (5), 408-17.
6. Stass, H.; Kubitza, D., Pharmacokinetics and elimination of moxifloxacin after oral and intravenous administration in man. *J Antimicrob Chemother* **1999,** 43 Suppl B, 83-90.
7. Fujino, H.; J., K.; Yamada, Y.; Kanda, H.; Kimata, H., Studies on the Metabolic Fate of NK-104, a New Inhibitor of HMG-CoA Reductase (4): Interspecies Variation in the Laboratory Animals and Humans. *Xenobio. Metabol. and Dispos.* **1999,** 14, (2), 79-91.
8. van Heiningen, P. N.; Hatorp, V.; Kramer Nielsen, K.; Hansen, K. T.; van Lier, J. J.; De Merbel, N. C.; Oosterhuis, B.; Jonkman, J. H., Absorption, metabolism and excretion of a single oral dose of (14)C-repaglinide during repaglinide multiple dosing. *Eur J Clin Pharmacol* **1999,** 55, (7), 521-5.
9. Stangier, J.; Schmid, J.; Turck, D.; Switek, H.; Verhagen, A.; Peeters, P. A.; van Marle, S. P.; Tamminga, W. J.; Sollie, F. A.; Jonkman, J. H., Absorption, metabolism, and excretion of intravenously and orally administered [14C]telmisartan in healthy volunteers. *J Clin Pharmacol* **2000,** 40, (12 Pt 1), 1312-22.
10. Ericsson, H.; Hamren, B.; Bergstrand, S.; Elebring, M.; Fryklund, L.; Heijer, M.; Ohman, K. P., Pharmacokinetics and metabolism of tesaglitazar, a novel dual-acting peroxisome proliferator-activated receptor alpha/gamma agonist, after a single oral and intravenous dose in humans. *Drug Metab Dispos* **2004,** 32, (9), 923-9.
